# *MicroRNA 218* Mediates the Effects of *Tbx5a* Over-Expression on Zebrafish Heart Development

**DOI:** 10.1371/journal.pone.0050536

**Published:** 2012-11-30

**Authors:** Elena Chiavacci, Luca Dolfi, Lorena Verduci, Francesco Meghini, Gaia Gestri, Alberto Mercatanti Monica Evangelista, Stephen W. Wilson, Federico Cremisi, Letizia Pitto

**Affiliations:** 1 Institute of Clinical Physiology, CNR, Pisa, Italy; 2 Department of Cell and Developmental Biology, University College London, London, United Kingdom; 3 Scuola Normale Superiore, Pisa, Italy; Mayo Clinic, United States of America

## Abstract

*tbx5*, a member of the T-box gene family, encodes one of the key transcription factors mediating vertebrate heart development. Tbx5 function in heart development appears to be exquisitely sensitive to gene dosage, since both haploinsufficiency and gene duplication generate the cardiac abnormalities associated with Holt−Oram syndrome (HOS), a highly penetrant autosomal dominant disease characterized by congenital heart defects of varying severity and upper limb malformation. It is suggested that tight integration of microRNAs and transcription factors into the cardiac genetic circuitry provides a rich and robust array of regulatory interactions to control cardiac gene expression. Based on these considerations, we performed an *in silico* screening to identify microRNAs embedded in genes highly sensitive to Tbx5 dosage. Among the identified microRNAs, we focused our attention on *miR-218-1* that, together with its host gene, *slit2*, is involved in heart development. We found correlated expression of *tbx5* and *miR-218* during cardiomyocyte differentiation of mouse P19CL6 cells. In zebrafish embryos, we show that both Tbx5 and *miR-218* dysregulation have a severe impact on heart development, affecting early heart morphogenesis. Interestingly, down-regulation of *miR-218* is able to rescue the heart defects generated by *tbx5* over-expression supporting the notion that *miR-218* is a crucial mediator of Tbx5 in heart development and suggesting its possible involvement in the onset of heart malformations.

## Introduction

The formation of the mature vertebrate heart with separated chambers and valves involves a complex orchestration of gene expression. Numerous genes are critical for cardiac morphogenesis, although their exact functions and their integration with other cardiac regulators is poorly understood [1].

The T-box gene *tbx5* encodes a key transcription factor for vertebrate heart development [2], [3]. Tbx5 function in the heart is gene dosage sensitive, as both haploinsufficiency and gene duplication give rise to Holt−Oram syndrome (HOS). HOS is a highly penetrant autosomal dominant disease characterized by congenital malformations of the heart and upper limbs, which are two sites of Tbx5 expression [4]–[7]. Nonetheless, the molecular mechanisms accounting for gene dosage sensitivity are not known. Mice heterozygous for mutations in Tbx5 display many of the phenotypic abnormalities of individuals with HOS [8], [9]. Comparable defects are seen in the zebrafish Tbx5 mutant *heartstrings*, suggesting that Tbx5 expression and function have been conserved throughout vertebrate evolution [10], [11]. In the murine model of HOS, gene expression profiling of a *tbx5* allelic series demonstrated that Tbx5 could regulate hundreds of downstream genes [8]. Examination of the expression dynamics of mouse genes regulated by Tbx5 indicates that Tbx5 can act via an array of independent mechanisms, some of which include direct DNA binding as has been shown for *Gja5* [9], and others via indirect mechanisms that may involve complex regulatory networks.

Several genes regulated by mouse Tbx5 encode transcription factors (TFs), or are involved in transcriptional regulation suggesting that HOS might in part be the consequence of “misregulation of regulators”. Besides TFs, microRNAs (miRNAs) play key roles in heart development and cardiac diseases [12]–[14]. TFs and miRNAs comprise two major layers of gene regulatory networks with strictly interconnected activities: TFs control miRNA expression and many miRNA targets are TFs. There is increasing evidence that TFs and miRNAs can work cooperatively through mutual cross-regulation [15]. Starting from these considerations, we performed an *in silico* screening to identify miRNAs embedded in genes highly sensitive to Tbx5 dosage. We focused our attention on *miR-218-1* that, together with its host gene *slit2*, is involved in heart development [16]. We confirmed a correlation between *tbx5* and *miR-218* expression and showed that alterations of *miR-218* expression have a significant impact on zebrafish heart development. Interestingly, down-regulation of *miR-218* is able to rescue most of the defects generated by Tbx5 over-expression, demonstrating the pivotal role of *miR-218* in mediating the effects of Tbx5 dosage on heart development. These data support the idea that a miRNAs/Tbx5 regulatory circuit is crucial in cardiac morphogenesis.

## Results

### Identification of Tbx5-modulated miRNAs

To identify miRNAs modulated by Tbx5, we developed a bioinformatic tool to search for miRNAs within introns of Tbx5-controlled genes (Fig. S1). As a source of Tbx5 targets, we used genes identified by microarray analysis of a conditional mouse allelic series of *tbx5* [8] and of a mouse 1H cell line infected with adenovirus expressing *tbx5* [17]. Four miRNAs were identified: *miR-218-1*, *miR-678*, *mir-719* and *miR-335* (Table 1). We focused our attention on *miR-218* since: i) it is conserved from human to zebrafish (www.mirbase.org); ii) the *miR-218-1* host gene, *slit2,* is highly sensitive to Tbx5 mis-expression [8]; iii) the secreted Slit ligands, together with their Robo receptors, contribute to the control of oriented cell tissue growth during chamber morphogenesis of the mammalian heart [18]; iv) Slit/*miR-218*/Robo are part of a regulatory loop required during heart tube formation in zebrafish [16].

**Table 1:** miRNAs identified by bioinformatic approach within introns of Tbx5-modulated genes.

Microarray	Gene symbol	miRNA	Tbx5 control	Proposed biological function
Mori/Plageman	Mest	miR-335	positive	Trabeculation, mouse embryonic cardiac expression [54]
Mori	Nupl 1	miR-719	negative	Nuclear pore complex
Mori	Slit 2	miR-218-1	positive	Secreted negative regulator of axonal extension
Mori	Hrmtl 1	miR-678	positive	S-adenosylmethionine-dependent methyltransferase activity

### tbx5 and miR-218 are Co-expressed in Mouse Tissues and in Cardiomyocyte Differentiation of P19CL6 Cells

We compared *tbx5*, *slit2* and *miR-218* expression in newborn mouse lung, liver, brain, aorta, skeletal muscle and heart. *slit3* expression was also analyzed since its host miRNA, *miR-218*-2, cannot be separately quantified because it is identical to *miR-218-1*. In agreement with the literature [19], we observed tight co-expression of *slit2* and *miR-218*, and a general correlation among *tbx5*, *slit2*, *slit3* and *miR-218* expression (Fig. S2).

**Figure 1:**
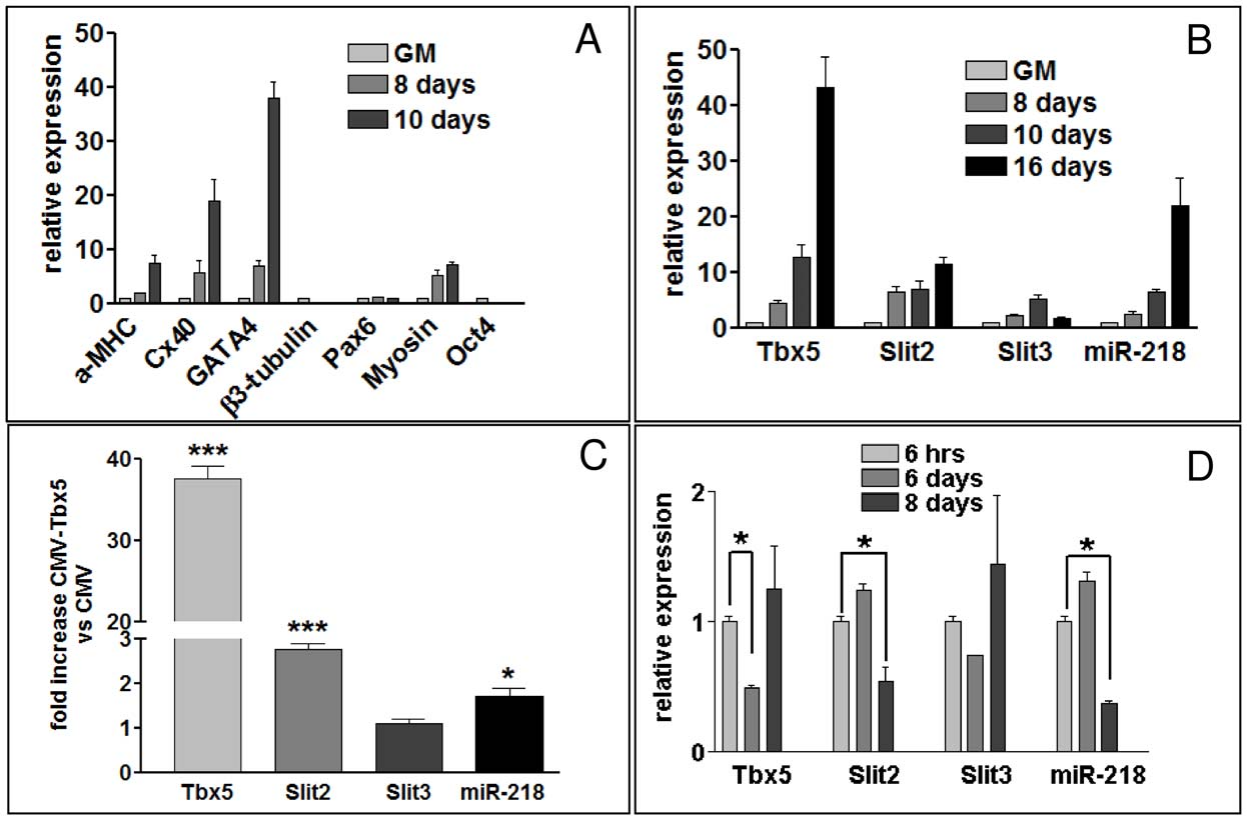
tbx5 and miR-218 are co-expressed in cardiomyocyte differentiation of P19CL6 cells. A, qRT-PCR analysis of cardiac (α-MHC, Cx40, GATA4), muscle (Myosin), neural (Pax6, β-3-tubulin) and pluripotency (Oct4) markers in P19CL6 differentiating cells (8,10 days) compared to P19CL6 in growth medium (GM). B, qRT-PCR analysis of t*bx5*, *slit2*, *slit3* and *miR-218* relative expression in either expanding (GM) or differentiating (8,10,12 days) P19CL6 cells. (C-D), P19CL6 differentiating cells, 48 h after CMV-Tbx5 transfection compared to cells transfected with empty vector (C) or different times after Tbx5-siRNA transfection compared with cells transfected with siRNA-Ct (D). The timing course of the silencing experiment in (D) is described in the “Cell culture and transfection” section of methods. Results are standardized against GAPDH for genes, and against U6 for miRNAs.

To assess whether there are functional regulatory interactions among Tbx5, *Slit2* and *miR-218*, we first examined these genes in an *in vitro* model for cardiomyocyte differentiation. P19CL6 cells proliferate in growth medium (GM) and differentiate into beating cardiomyocytes in differentiation medium (DM) [20], [21]. P19CL6 increased the expression of cardiac differentiation markers such as GATA4, α-MHC, CX40 and decreased the expression of the marker of pluripotency Oct4 after a few days of culture in DM, compared to cells maintained in GM (Fig. 1A). A progressive increase in *tbx5* expression was also observed (Fig. 1B), which was paralleled by an increase in *slit2*, *slit3* and *miR-218* transcripts. To show that the *slit*/*miR-218* increase was at least partially dependent on Tbx5, *tbx5* was up- or down-regulated by transfecting P19CL6 cells with a *tbx5*-carrying expression vector (CMV-Tbx5), or with a siRNA mix directed against *tbx5*, respectively. Tbx5 over-expression tripled *slit2* expression, almost doubled *miR-218* expression and had no effect on *slit3* expression (Fig. 1C). On the other hand, *tbx5* silencing, the effect of which was highest 2 days after silencing (6th day in culture, see Methods), caused significant reduction of *slit2* and *miR-218* expression 4 days after transfection (8th day in culture, Fig. 1D). Pre-miRNA 218-1 expression paralleled the increase in *miR-218* level during cardiomyocyte differentiation (Fig. S3A) and after Tbx5 modulation (Fig. S3B). Moreover, the transfection of a siRNA mix against *Slit2* cut the level of *Slit2* by half without affecting *miR-218* expression, supporting the idea that *miR-218* expression depends on the regulation of *Slit2* transcription rather than on its translation. Overall these results suggest that the expression of *Slit2* and its embedded microRNA *miR-218-1* are modulated by Tbx5 during cardiomyocyte differentiation.

### miR-218a Over-expression Affects Zebrafish Heart Development

To analyze the role of *miR-218* in heart development, we decided to use zebrafish since this model is particularly informative for studying cardiac early patterning networks due to its relatively simple two-chambered heart coupled with its ability to develop even in the absence of a functioning heart. Moreover, various data derived from Tbx5 knock-down experiments in zebrafish [10], [11] have revealed developmental defects of the heart and limbs comparable to the defects observed in human with Tbx5 mutations [4], [22] or in Tbx5 knock-down mice [9], [23], suggesting that the functional role of this crucial transcription factor is evolutionarily conserved.

**Figure 2:**
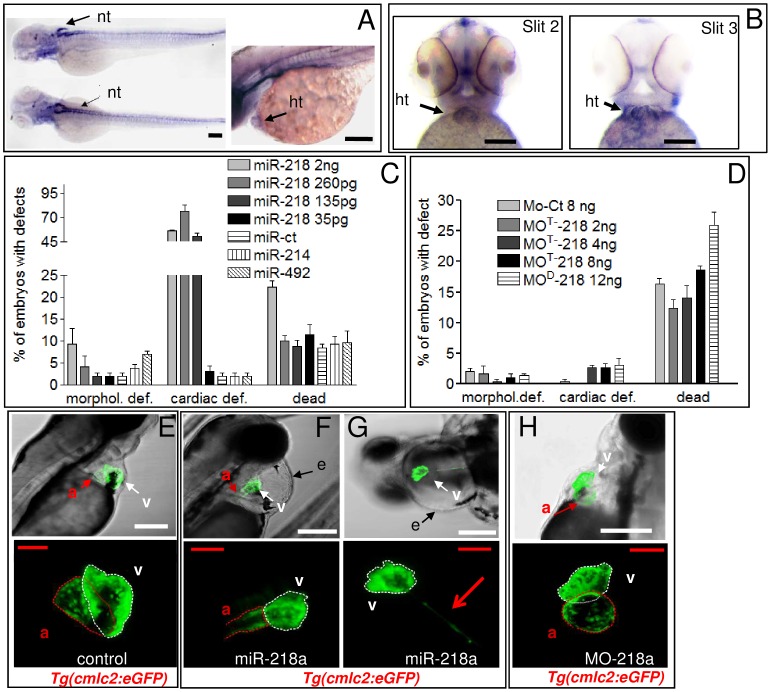
miR-218 over-expression affects cardiac development. A, *miR-218*a ISH of 72 hpf embryos. B, *slit2* and s*lit3* ISH of 48 hpf embryos. nt, neural tube; ht, heart. C,D, phenotypes induced at 72 hpf by increasing doses of *miR-218* mimic (C) or MO^M^/MO^D^-218 (D) injection. The percentage of embryos with the indicated defects was averaged across multiple independent experiments carried out in double blind. The total numbers of embryos analyzed were as follows: Ct miRNA (1 ng) n = 293; miR-214 mimic (1 ng) n = 104; miR-492 mimic (1 ng) n = 103; *miR-218* mimic (35 pg) n = 107; *miR-218* mimic (135 pg) n = 180; *miR-218* mimic (260 pg) n = 318; *miR-218* mimic (2 ng) n = 180; MO-Ct (8 ng) n = 207; MO^D^-218 (12 ng) n = 323; MO^M^-218 (2 ng) n = 112; MO^M^-218 (4 ng) n = 165; MO^M^-218 (8 ng) n = 182. E-H, phenotypic analysis of *miR-218*a misregulation in *Tg(cmlc2:eGFP)* embryos. Confocal images of representative transgenic embryos showing the presence or the absence of pericardial edema (e;top) and heart morphology (bottom). F,G examples of heart defects with different degrees of severity. a, atrium, v, ventricle, e, cardiac edema. Dotted lines encircle ventricle (white) or atrium (red). Red arrow in G (bottom panel) indicates a shrunken, elongated ventricle typical of the heartstring phenotype. Scale bars: white or black 100 µm, red 25 µm.

In zebrafish, as in mammals, two isoforms of *miR-218*, *miR-218a-1* and *miR-218a-2,* are embedded in *slit3* and *slit2* genes, respectively. A third genomic copy of this miRNA, *miR-218b*, is present in fish. However *miR-218b*, an intergenic miRNA, has very low expression, suggesting that its contribution to the global *miR-218* level might be irrelevant [16]. *miR-218a1/2*, *slit2* and *slit3* are highly expressed in zebrafish neural tissue (Fig. 2A, [24], [25]) and Fig. 2B [26]–[28]. In cardiac tissue, *slit2* and *slit3* are clearly detectable at 48 hpf (Fig. 2B), while the expression of *miR-218a1/2* is barely detectable by in situ hybridization (ISH) up to 48 hpf (not shown) but is visible around 72 hpf (Fig. 2A).

To over-express *miR-218*a1/2, we injected double stranded RNA oligonucleotide with a *miR-218a* sequence (*miR-218*a mimic) in *Tg(cmlc2:eGFP*) embryos. Injection of 260, 135 and 35 pg of *miR-218*a mimic generated embryos with cardiac defects in a dose-dependent way (Fig. 2C). At the highest dose (2 ng in Fig. 2C), we observed a slight decrease in embryos with cardiac defects, which was balanced by the increased number of dead embryos and, to a lesser extent, of embryos with different morphological defects. As specificity controls, 2 ng of either *miR-492*, a control miRNA, that is not annotated in zebrafish, or *miR-214,* which is not heart-specific, were injected without generating embryos with cardiac defects in significant percentages (Fig. 2C). Conversely, in *miR-218a* over-expressing embryos we found different cardiac defects accounting for the ratios shown in Fig. 2C: hearts failing to complete looping, ventricles showing very irregular walls, and atria that were strongly reduced and sometimes stretched to a thin “string-like” morphology (Fig. 2F,G). To down regulate *miR-218a*, we injected either a morpholino targeting the mature form of *miR-218a* (MO^M^-218), or a longer morpholino also targeting the Drosha cleavage site of pre-*miR-218*a (MO^D^-218, see Methods and [16]). The knockdown efficiency of these morpholinos was confirmed by their ability to down-regulate mature *miR-218* (Fig. S4A), and to rescue the phenotype caused by *miR-218a* over-expression (Fig. S4B). Nonetheless, injection of either MO^D^-218 or MO^M^-218 (not shown) generated no cardiac edema or cardiac defects even at very high dose (12 ng) in any of the genetic backgrounds we tested (Fig. 2D,H). This is consistent with the extremely low level of *miR-218a* during early stages of development (data not shown and [16], [29]).

Injection of *miR-218a* mimic or MOs-218 in *Tg(flk1:eGFP*), which express GFP driven by the endothelial-specific enhancer of *flk1,* allowed a direct visualization of vascular integrity. As previously reported [16], MO-218 microinjection did not cause gross alteration in vascular structures (Fig. S5C) nor the hemorrhagic events described in mice after *miR-218a* knock down [19]. *miR-218a* over-expression did not affect vessel morphology either (Fig. S5B), supporting the idea that in zebrafish *miR-218a* does not overtly influence the organization of blood vessels during embryogenesis.

**Figure 3:**
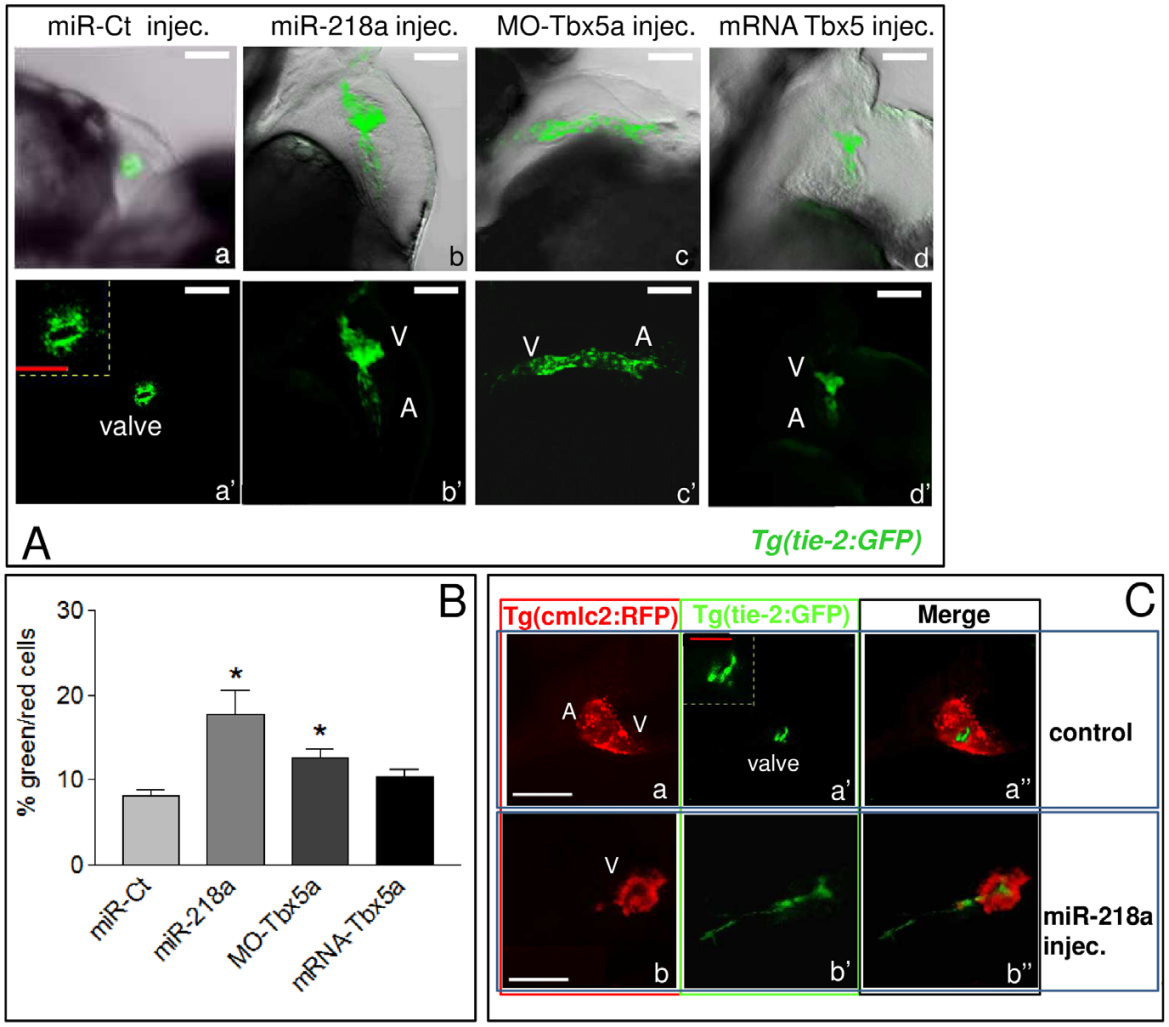
miR-218a over-expression leads to the expansion of tie-2 expression. A, confocal images of 72 hpf *Tg(tie-2:GFP)* embryos injected with 260 ng of control miRNA (a,a′), 260 ng of *miR-218*a mimic (b,b′), 2 ng of MO-Tbx5a (c,c′) or 100 pg of mRNA Tbx5a (d,d′). A, magnification of the control valve is shown in the inset in panel a′. Labels: A, atrium, V, ventricle. B, FACS analysis of cells dissociated from 72 hpf *Tg(tie-2:GFP-cmlc2:eRFP)* embryos injected as described in A. C, confocal images of 72 hpf *Tg(tie-2:GFP-cmlc2:eRFP)* embryos injected with 260 ng of miR-Ct (top) or with *miR-218*a mimic (bottom). The control valve is magnified in the inset in panel a′. White scale bars: 100 µm, red scale bars 25 µm.

Finally, we analyzed the expression of some cardiac markers in embryos over-expressing *miR-218a*. Despite the strong cardiac morphological alterations, *miR-218a* over-expressing embryos showed normal expression of the ventricular myosin heavy chain and the atrial myosin heavy chain (Fig. S6), as previously described in Tbx5 (*hst)* mutants [10]. It has been shown that Tbx5 activity is crucial for the specification of the AV boundary and valve formation [30]. To verify whether *miR-218* over-expression can affect cardiac valve development, we analyzed the expression of the *tie-2* gene, a member of the Tie family of tyrosine kinase receptors, which is expressed mainly in endothelial cells [31] and is up regulated during atrio-ventricular canal differentiation [32], [33]. In hemizygous *Tg(tie-2:GFP)* embryos, the transgene has low expression [34] and it is easy detectable only in the atrio-ventricular canal thus allowing to visualize the valve tissues (Fig. 3A, a,a′). Hemizygous *Tg(tie-2:GFP)* embryos injected with *miR-218*a showed an increase in Tie-2 expressing cells in the ventricle and atrium (Fig. 3A, b,b′ and Fig. 3B,C). A similar Tie-2 dysregulation was observed in hemizygous *Tg(tie-2:GFP)* embryos after Tbx5 misexpression (Fig. 3A, c,c′,d,d′ and Fig. 3B).

All together these data indicate that correct expression of *miR-218* is crucial for proper cardiac development.

### miR-218 Over-expression Decreases the Migration of Myocardial Precursors

**Figure 4:**
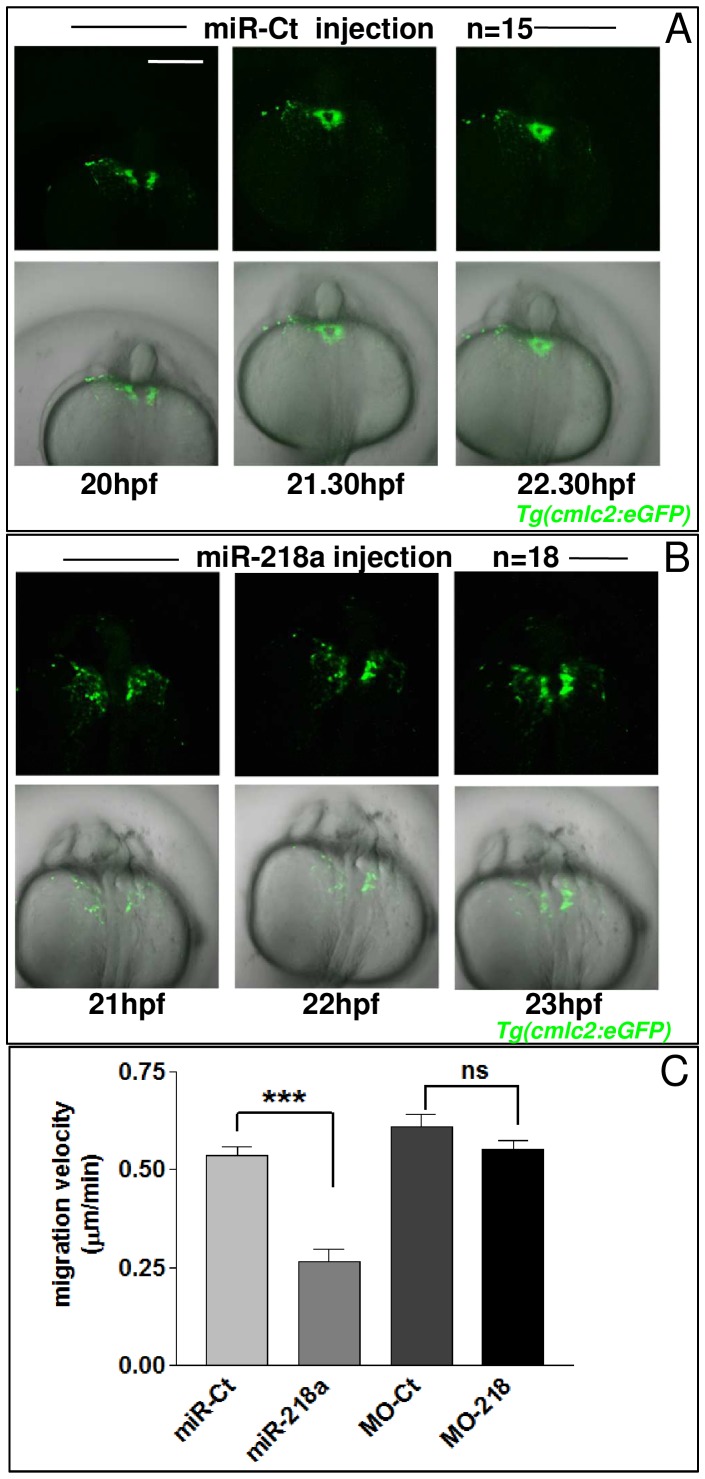
miR-218 over-expression causes a delay in early heart field migration. A,B, images of *Tg(cmlc2:eGFP)* embryos injected with 260 pg of miR-Ct (A) or with 260 pg of *miR-218*a mimic (B) at different times of development. Dorsal view, anterior at the bottom. After confocal analysis, embryos were left to develop until 72 hpf when they were screened for the presence of edema. White scale bars: 150 µm. C, migration velocities of myocardial *Tg(cmlc2:eGFP)* cells as quantified from time-lapse images. Five embryos for each experiment were analyzed.

Recent data have shown that, in zebrafish, delayed heart field migration was caused by either *miR-218a* reduction or by silencing of Robo1, an established target of *miR-218a* [16], [19]. In line with data showing *robo1* as target of *miR-218a*, over-expression of *miR-218a* significantly reduces the translational rate of a reporter construct (sensor), carrying GFP coding sequence upstream of *robo1* 3′UTR (Fig. S7). Therefore, we decided to verify whether *miR-218a* over-expression might modify the migration of bilateral heart field cells to the midline. *miR-218a* mimic was injected in *Tg(cmlc2:eGFP)* embryos and the migration of the GFP-expressing cells was followed by confocal analysis. Myocardial cells migrated more slowly in *miR-218a* than in *miR-Ct* injected embryos (Fig. 4). Even after injection of high *miR-218* doses, cardia bifida was never observed, suggesting that *miR-218* over-expression slows down but does not arrest the migration of cardiomyocytes to the midline. On the contrary, MO^D^-218 injection did not affect cmlc2-positive cells migration (Fig. 4C), further confirming that reducing the level of this miRNA during the first hours of development has no overt effects on heart development.

These data show that the cardiac defects observed in *miR-218a* over-expressing embryos might be at least in part related to defects in heart field migration.

### Tbx5 Over-expression can be Rescued by Down-regulation of miR-218

**Figure 5:**
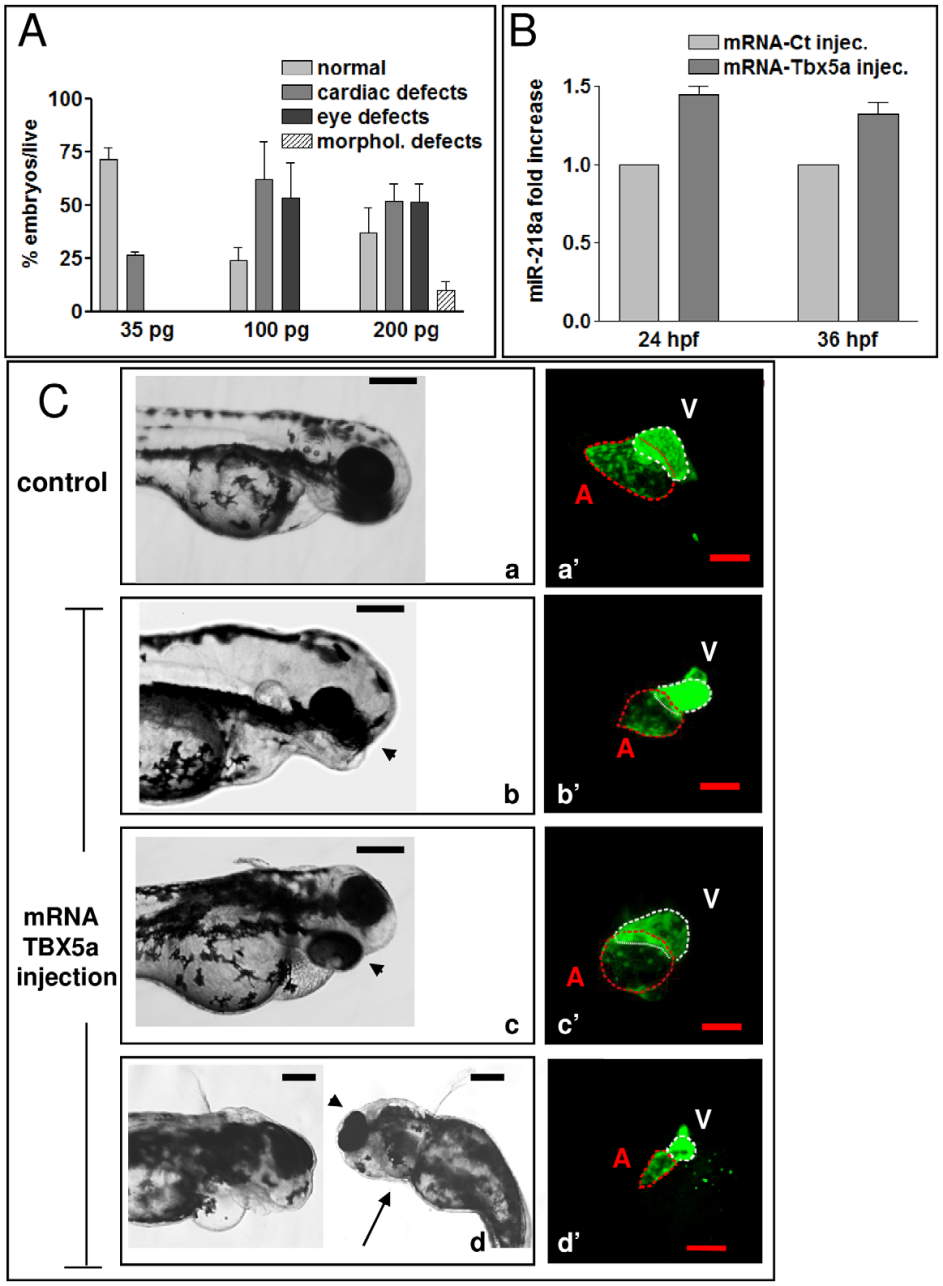
tbx5 over-expression causes eye, cardiac and fin defects. A, phenotypes generated by increasing doses of *tbx5a* mRNA. The percentage of embryos with the indicated defects was averaged across multiple independent experiments. The total number of embryos analyzed was as follows: mRNA-Tbx5a (35 pg) n = 48; mRNA-Tbx5a (100 pg) n = 199; mRNA-Tbx5a (200 pg) n = 131;. B, qRT-PCR analysis of *miR-218*a relative expression in 24 and 34 hpf embryos injected with 100 pg of *tbx5a* mRNA compared with embryos injected with 100 pg of GFP mRNA. C, phase-contrast and confocal images of representative transgenic *Tg(cmlc2:eGFP)* embryos at 72 hpf showing eye, heart and fin morphological defects induced by the injection of 100 pg (b,b′–c-c′) or of 200 pg (d,d′) of *tbx5a* mRNA. Arrowheads indicate eye alteration, arrows show fin absence. Labels: A, atrium, V, ventricle. Dotted lines encircle ventricle (white) and atrium (red). Scale bars: black 100 µm, red 25 µm.

**Figure 6:**
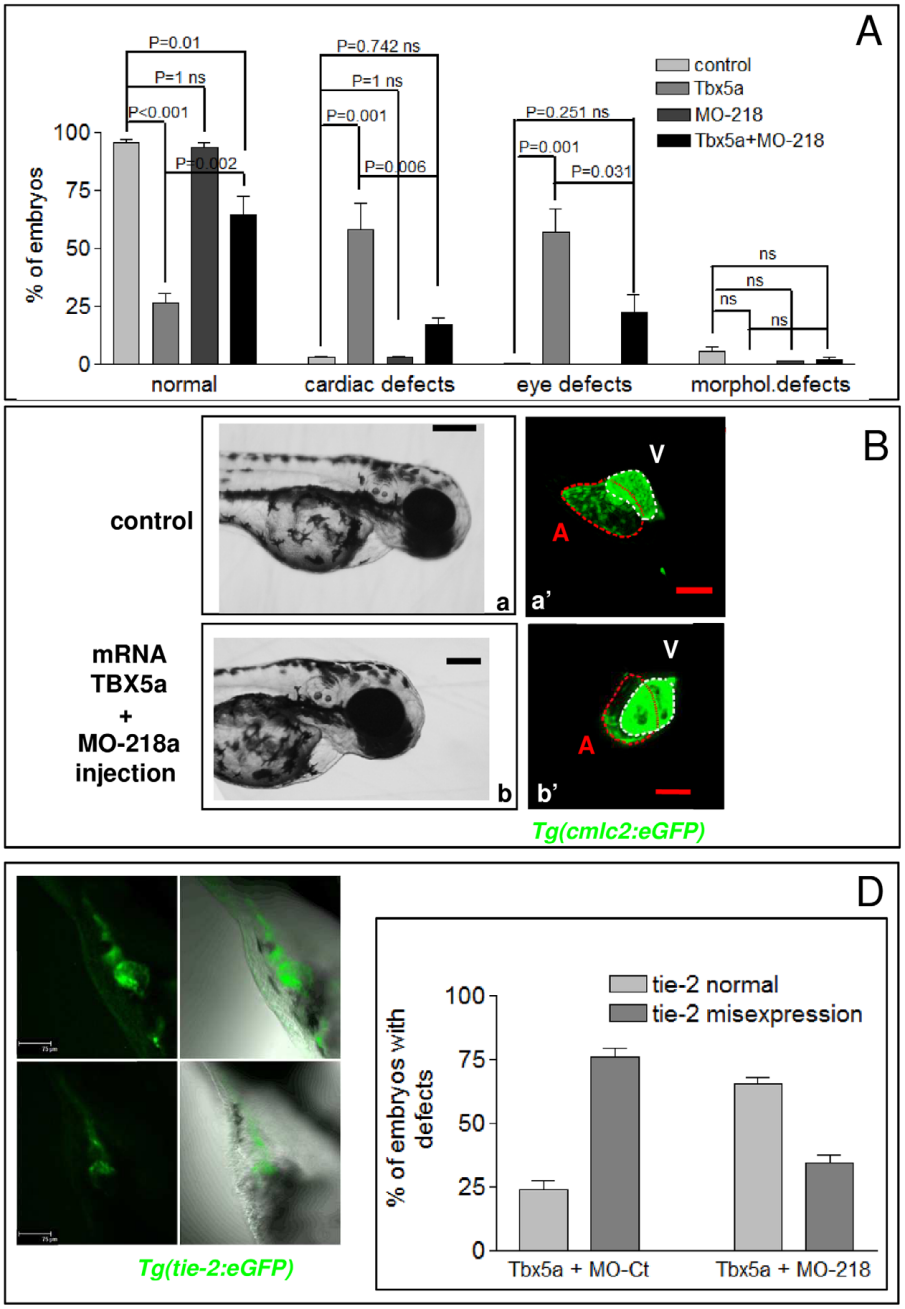
Down-regulation of miR-218 can rescue the defects generated by tbx5 over-expression. A, quantification of the phenotypes induced by the injection of 100 pg of *tbx5a* mRNA (n = 199), 8 ng of MO^D^-218 (n = 182) or by the co-injection of 100 pg of *tbx5a* mRNA and 8 ng of MO^D^-218 (n = 241). As control, non injected embryos were quantified. Each experimental point in the graph represents the mean ± SE of at least three independent experiments. Comparisons between groups were performed by one-way analysis of variance, followed by Bonferroni’s post-hoc for multiple comparisons. B, phase-contrast and confocal images of representative transgenic *Tg(cmlc2:eGFP)* embryos at 72 hpf comparing the phenotype of a control embryo (upper panels) to the rescued phenotype generated by the co-injection of 100 pg of *tbx5a* mRNA and 8 ng of MO^D^-218 (lower panels). C, quantification of *tie-2* mis-expression in 72 hpf *Tg(tie-2:GFP)* embryos after the co-injection of *tbx5a* mRNA (100 pg) and MO-Ct (8 ng, n = 60) or of *tbx5a* mRNA (100 pg) and MO^D^-218 (8 ng, n = 62). D, Confocal images of representative 72 hpf *Tg(tie-2:GFP)* embryos co-injected with *tbx5a* mRNA and MO-Ct (a-a′) or with *tbx5a* mRNA and MO^D^-218 (b-b′). Labels: A, atrium, V, ventricle. Scale bars: black 100 µm, red 25 µm.

To further investigate the functional interaction between Tbx5 and *miR-218a*, we assessed whether the morphological alterations generated by Tbx5 misexpression might be compensated for the modulation of *miR-218a*. We reasoned that if the phenotype induced by Tbx5 over-expression was due, at least in part, to increased expression of *miR-218*, the co-injection of MO-218 should rescue the Tbx5 gain of function phenotype. To test this hypothesis, Tbx5a was over-expressed by injecting its mRNA into the cytoplasm of one–cell-stage embryos. Tbx5 over-expression affects heart development in humans [35], [36],mice [37] and chicks [5], while no reports describe the effect of Tbx5 over-expression in zebrafish. Over-expression of *tbx5a* mRNA induced a range of cardiac defects, from cardiac edema in 25% of embryos injected with low doses of mRNA (35 pg), to mild cases of looping defects or absence of looping and alteration of chamber morphology in 60% of embryos injected with higher doses (100 pg, Fig. 5A and C, panels b′–d′). Pectoral fins were occasionally asymmetric in embryos injected with 200 pg of *tbx5a* transcript (Fig. 5C, panel d). *tbx5a* mRNA injection also caused eye morphology malformations ranging from asymmetrically positioned eyes, fusion of eyes or reduction/absence of eyes (Fig. 5C, panels b,c). None of these defects was observed at significant percentages in hundreds of embryos injected with comparable doses of GFP mRNA (not shown). Increased *miR-218a* expression was observed as a consequence of Tbx5 up regulation (Fig. 5B). Co-injection of 8 ng of either MO^D^-218a or MO^M^-218a along with 100 pg of *tbx5a* mRNA restored normal heart morphology and looping in a high percentage of injected embryos and reduced the number of embryos with eye defects as compared to control-injected embryos (Fig. 6A and B, panels b,b′). Co-injection of MO-218 was also able to counteract the *tie-2* expansion observed in Tbx5 dysregulated embryos (Fig. 6 C,D). This result strengthened our hypothesis that the effect of Tbx5 over-expression on heart development might, at least in part, be be mediated by *miR-218*.

The apparent lack of phenotype that we observed after MO-218a injection (Fig. 2D,I) and the very low expression of *miR-218* at early developmental stages, suggest that decreased *miR-218a* should not contribute to the phenotype generated by Tbx5 knockdown. To verify this assumption we downregulated Tbx5 using a morpholino directed against the *tbx5a* translational start site (MO-Tbx5a). The effectiveness of this morpholino was confirmed by the injection of GFP-carrying a chimeric target (Fig. S8A,B) and its specificity was confirmed by rescuing the MO-Tbx5a phenotype co-injecting *in vitro* transcribed Tbx5a-mRNA (not shown). Tg(*cmlc2*:GFP) embryos injected with MO-Tbx5a showed the heart and limb defects characteristic of the well described *heartstrings* phenotype (Fig. S8E,G and [10], [11]). In addition to *tbx5a*, a second *tbx5* isoform has been recently identified in zebrafish, *tbx5b*, which has lost the characteristic forelimb/pectoral fin expression of *tbx5* genes [38]. Consequently, we also designed a morpholino against this *tbx5* isoform, MO-Tbx5b (Fig. S8C,D). Similarly to MO-Tbx5a injection, MO-Tbx5b injection caused looping defects and stretched cardiac chambers, but it did not induce Tie-2 mis-expression and lack of fins (Fig. S8F). For these reasons and since the penetrance of the MO-Tbx5b phenotype was much lower compared to MO-Tbx5a (compare Fig. S8G,H) we chose MO-Tbx5a for further analysis. Co-injection of mimic *miR-218a* did not rescue the cardiac edema and looping defects generated by MO-Tbx5a (Fig. S9A). Instead, the co-injection increased both the frequency and the severity of the mutant phenotype, doubling the number of *heartstring*-like embryos (Fig. S9A) and the number of embryos that showed a particularly extended edema at 48 hpf (Fig. S9B). This synergism was further demonstrated by MO-Tbx5a and *miR-218a* mimic co-injection in sub-phenotypic doses. In fact, zebrafish embryos tolerated the injection of either 35 pg of *miR-218a* or 0,5 ng of MO-Tbx5a well and only rarely revealed heart looping defects as compared to un-injected siblings (Fig. 2A and Fig. S9C,D). However, the injection of both MO-Tbx5a and *miR-218a* mimic at these doses dramatically increased the number of embryos with looping defects (Fig. S9C,E).

As a whole, these data support a functional interaction between Tbx5 and *miR-218a* in heart morphogenesis.

## Discussion

Our data show that *miR-218* is part of a regulatory circuit through which Tbx5 controls heart morphogenesis. Previous studies in mice identified *slit2*, which encodes *miR-218-1* within one of its introns, as one of the genes highly sensitive to Tbx5 dosage [8]. Moreover, the coordinate expression of *miR-218-1* and its host genes has been largely documented both in physiological (mouse development [19]) and pathological (cancer progression [39], [40]) conditions. We showed a functional relation between Tbx5, Slit2 and *miR-218* in P19CL6 cells in which a progressive increase of Tbx5, Slit2 and *miR-218* expression was observed during cardiomyocyte differentiation. Moreover, we showed that Tbx5 deregulation affects *miR-218* expression. Tbx5 might regulate *slit2* expression directly or indirectly. However the presence of T-box consensus sequences upstream of both mouse and fish *slit2* transcription start site, as identified by the Transfac program (http://www.biobase-international.com; not shown), supports the hypothesis that Tbx5 might directly bind to and activate the *slit2* promoter. To demonstrate that the Tbx5/*miR-218* regulatory circuit is also functional during development, we used the zebrafish as a model system. Through functional assays in zebrafish, we showed that either over-expression or down-regulation of Tbx5 affects heart morphogenesis. In line with the hypothesis that *miR-218* might be a Tbx5 effector, we demonstrated that *miR-218*a deregulation generates cardiac defects (Fig. 2A). As in zebrafish the expression level of *miR-218a* is extremely low during the first stages of development (our observation is supported also by the data of Fish et al. [16] and by data regarding miRNA microarray of Thatcher et al. [29], the introduction of very low amounts of *miR-218a* mimic into embryos at the one- or two-cell stage generated a severe cardiac phenotype. *miR-218a* over-expressing hearts were not able to complete the looping process, showed marked alteration of the cardiac chamber morphology and mis-expression of a marker of valve cardiac tissues. A large pericardial edema was visible after 48 hpf. The frequency and severity of all these cardiac phenotypes were dose-dependent (Figs. 2 and 3). Our data suggest that at least part of these morphological anomalies may be due to a reduced migration rate of myocardial cells (Fig. 4). Conversely, *miR-218a* down regulation, even through injection of high doses of two different morpholinos, did not affect heart development.

Since Tbx5 and Slit2 are both expressed in myocardial cells [16] we hypothesized that early Tbx5 over-expression might cause heart malformation through early activation of *miR-218a* and silencing of the target genes of this miRNA. This hypothesis is supported by the observation that *miR-218* down-regulation by MO-218a injection rescues the effects of Tbx5 over-expression (Fig. 6). *robo1* has been identified as a target of *miR-218* in many different organs and tissues [19], [39], [41]. Fish et al [16] showed that *robo1* is a *miR-218*a direct target and that early *robo1* down-regulation by morpholino injection in zebrafish embryos induces severe pericardial edema and heart defects caused by reduced migration rate of endocardial cells. Thus, *robo1* is a candidate gene through which Tbx5 and *miR-218a* early over-expression affects heart development. Fish et al [16]reported that *miR-218*a down regulation, performed by the microinjection of one of the two morpholinos that we used in our study, causes severe cardiac defects and cardiac edema through reduced migration of endocardial and myocardial cells. This is inconsistent with both our data and with the results of Fish et al. [16] who observed that Robo1 knock-down generates the same phenotype, because the same authors also showed that Robo1 is targeted by *miR-218*. In fact, an effect of *miR-218*a on the migration of endocardial and myocardial cells is not expected, as *miR-218*a seems not to be substantially expressed before 24 hpf [16], [29], when the fusion of migrating cardiac cells is about to be completed. We speculate that the timing of *miR-218*a upregulation during heart development is crucial for heart morphogenesis. *miR-218* and Robo1 are supposed to be upregulated and downregulated, respectively, when myocardial cell migration is about to end. In this view, Tbx5 mis-expression by mRNA microinjection at the one-cell stage might speed up the up-regulation of *miR-218* and reduce the migration of myocardial cells precociously, which in turn might affect heart morphogenesis by impairing the correct interaction between myocardial and endocardial cells.

A negative role of *miR-218* on cell migration has been highlighted in different biological contexts. In mice, it has been shown that *miR-218* regulates vascular patterning by modulating Slit-Robo signaling [19]. The authors showed that *miR-218*-driven repression of Robo1/2 and of heparan sulfate proteoglycans (HSPGs), which are proteins essential for Slit/Robo signaling, negatively affects endothelial cell (EC) migration. *miR-218* has also been shown to affect cancer progression by inhibiting tumor cell migration and metastasis via the repression of the Slit2/Robo1 pathway in gastric [40] and in nasopharyngeal [39] tumors, respectively. Therefore our data showing a migration delay of *cmlc2*-positive cardiac precursors in embryos over-expressing *miR-218* (Fig. 4), but not in embryos in which *miR-218* was down-regulated by MO-218 injection (Fig. 4C), are in line with these findings.

In the human heart, Tbx5 is expressed not only in the myocardium, but also throughout the embryonic epicardium and coronary vasculature. Using chick development as a model system, Hatcher et al. [42] showed that, *in vivo*, over-expression of biologically active Tbx5 inhibits proepicardial cell migration. Although we do not know whether the *slit2* is expressed in proepicardial cells, it is tempting to speculate that the up regulation of the Tbx5-*miR-218* circuit might also impact the proepicardial cell migration by targeting robo1 or other cell migration regulators such as Semaphorins, some members of this large class of molecules being predicted targets of *miR-218* (not shown).

Tbx5 over-expression affects heart development in different organisms. In humans, *tbx5* gene duplication produces cardiac abnormalities [35], [36]; in mice, persistent cardiac Tbx5 over-expression results in heart looping defects and abnormalities of early chamber development [37]. In chicks, Tbx5 over-expression determines a significant decrease in heart size and a marked decrease in ventricular trabeculation [5]. We also observed looping and cardiac chamber alterations in zebrafish after injection of *tbx5*a mRNA in embryos at one-cell stage. Unexpectedly, the severity of cardiac morphology defects was paralleled by the severity of eye defects (Fig. 5). Tbx5 is expressed in optic primordia from zebrafish to humans [43], [44] and its mis-expression has been shown to affect eye morphogenesis and the visual projection in chicks [45]. Moreover, ophthalmological examination of HOS patients revealed alteration of dorso-ventral polarity in developing eye vesicles [46]. However, our Tbx5 over-expressing embryos showed particularly severe eye defects such as asymmetrically positioned eyes, fusion of eyes, and even unilateral anophthalmia (Fig. 5). Comparable eye defects are observed in Brg1 over-expressing zebrafish embryos [47]. Recently the importance of the balance between Brg1, a member of the SWI/SNF chromatin-remodeling complex, and several cardiac transcription factors including Tbx5, has been demonstrated [48]. Since Brg1 is maternally expressed [47], the over-expression of Tbx5 might generate a strong imbalance between these two factors during the first hours of development, and this imbalance might be at the root of eye defects. At the moment we do not know how *miR-218* might also partially rescue eye defects. It is interesting to know that Pax2, that is negatively controlled by Tbx5 [45], is a predicted target of *miR-218*. Overall, these observations suggest that Tbx5 over-expression affects heart and eye development and that this might be at least partially mediated by *miR-218*. Our observation that MO-218 co-injection is able to ameliorate both heart and eye defects caused by Tbx5 injection is consistent with this model.

Our data suggest that the haplo-insufficiency of the Tbx5 gene, at the moment the most significant cause of HOS, does not impact heart and upper limb formation through *miR-218* misregulation. The simplest explanation for this might be that other key RNAs controlled by Tbx5 than *miR-218* might be necessary for heart morphogenesis by regulating mechanisms other than myocardial cell migration. This would still be consistent with the requirement of a tight *tbx5* gene dosage regulation in space and time for proper heart morphogenesis. More rarely, HOS has been associated with increased Tbx5 expression by partial chromosome duplication [6], [28] or mutation resulting in Tbx5 gain of function [49]. However advances in DNA sequencing technology also highlighted the potential role of non-coding variants in congenital malformations. Recent studies in mice uncovered three enhancers that collectively recapitulate the endogenous expression pattern of *tbx5* but that singularly have specific and compartmentalized expression in the heart and forelimbs [50]. Interestingly, a mutation in one of these enhancer sequences was identified in a cohort of non-syndromic patients and it has been shown to affect the enhancer activity in mice and zebrafish transgenic models. The population-wide frequency of this variant suggests that a significant number of congenital heart defects (CHD) associated with Tbx5 dysregulation might arise from non-coding mutations in Tbx5 heart enhancers effectively decoupling the heart and hand phenotypes of HOS syndrome. Therefore it is likely that modulation of Tbx5 in general, and over-expression of *miR-218* as a consequence of Tbx5 up-regulation in particular, might have a higher impact on CHD population than previously hypothesized.

Finally, our results highlight the potential advantage of using miRNAs as target molecules for heart disease therapies. Their potential to restore the expression of hundreds of dysregulated mRNAs to their pre-pathological level in one shot and, in so doing possibly reverse the disease, places miRNAs among the most exciting molecules for the development of new therapeutic strategies.

## Methods

### Bioinformatic Analysis

Ensembl (http://www.ensembl.org/index.html), was used to obtain information about chromosome location, position and segment of the selected genes. Ensembl is a joint scientific project between the European Bioinformatics Institute (EBI; http://www.ebi.ac.uk/clustalw/) and the Wellcome Trust Sanger Institute (http://www.sanger.ac.uk/). EBI provides a centralized resource with annotations on genomes of sequenced species and the Ensembl Perl API (Application Programming Interface) models for access to biological objects, such as genes and proteins. Moreover EBI allows the execution of Perl programs for retrieving data from a public database MySQL (http://www.mysql.it/). We generated two local databases, one for genes, and one for microRNAs. By applying the Perl program that uses Ensembl API, we compared the gene databases with the microRNA database using a standard database interface module for Perl.

*Reagents*Mature miRNA mimics (mmu-*miR-218*a, mmu-miR-492, mmu-miR-214 and miR-Ct) were synthesized by GenePharma (Shanghai, China), morpholinos (Gene Tools, LLC USA.), Lipofectamine 2000 TRIzol reagent, DNaseI amplification grade, SuperScript II reverse transcriptase, RNAse out, α-minimal Essential Medium (Invitrogen), HyPerFect Transfection Reagent, miRNeasy mini kit, miScript Reverse Transcription kit Quantitec Reverse Transcription kit and Quantifast SYBR Green PCR kit (QIAGEN, Milan, Italy), LightCycler 480 SYBR Green I Master, anti-DIG antibody-alkaline phosphatase Fab fragment, Blocking reagent, BM Purple and DIG-RNA labeling kit (Roche Diagnostic, Mannheim, Germany), DIG-labeled miRCURY LNA microRNA detectin probe (Exiqon), SP6 RNA polymerase, RNAse free DNAse I (Fermentas International Inc), Herculase DNA polymerase, (Stratagene), pGEM Teasy vector, (PROMEGA), Fetal Bovine Serum (Lonza), zebrafish diet (SDS, Dietex, France), Tetramisole (Sigma), mMESSAGE mMACHINE® Kit (Applied Biosystems).

### Zebrafish Lines

Current italian national rules: no approval needs to be given for research on zebrafish embryos. Wild-type AB and transgenic *Tg(flk1:eGFP), Tg(cmlc2:eGFP), Tg(cmlc2:eRFP)* and *Tg(tie-2:GFP)^s849^* transgenic lines were used in these studies. Zebrafish were raised and maintained under standard laboratory conditions (Westerfileld M zebrafish book) in a ZEBTEC Zebrafish Housing System (Tecniplast, Varese, Italy).

### Cell Culture and Transfection

P19CL6 cells were obtained from Dr.Antonio Baldini (Telethon Institute of Genetics and Medicine**,** Napoli, Italy). Cells were cultured in growth conditions as previously described [20], [21]. For differentiation conditions, growth medium was supplemented with 1% DMSO and the medium was replaceded every two days. Cells were grown at 37°C in a humidified atmosphere containing 6% CO2. The pCMV-Tbx5 plasmid expressing mouse Tbx5 (Kindly provided by Prof. Mona Nemer, Universitè d’Ottawa) or the pCMV empty vector as control were transfected using Lipofectamine 2000. To downregulate mouse Tbx5 the following siRNAs were designed according to previously identified criteria [51]: siRNA-Tbx5-1 (5′-CUGG ACCCGUUUGGACACAUU-3′/5′-UGUGUCCAAACGGGUCCAGUU-3′) and siRNA-Tbx5-2 (5′-GCCC CGAUUACACAUCGUGUU-3′/5′-CACGAUGUGUAAUCGGGGCUU-3′). To downregulate mouse *slit2* the following siRNAs were designed: siRNA-Slit2-1 (5′-GCUCACUCUGAACAAUAACUU-3′/5′-GU UAUUGUUCAGAGUGAGCUU-3′) and siRNA-Slit2-2 (5′-GGCUCAGAAGGAAAAGAAUUU-3′/5′-AUUCUUUUCCUUCUGAGCCUU-3′). For the silencing experiments in P19CL6, cells were transfected with control siRNA or a siRNA-Tbx5-1 and siRNA-Tbx5-2 mix in GM. 6 hrs after transfection the GM was substituted with the DM. Four days after the first round of transfection, the cells were transfected with the respective siRNA again. Cells were pelletted 6 h after the first transfection, 48 h and 96 h after the second round of transfection. SiRNAs were transfected using HiPerFect Transfection reagent.

### Quantitative Real Time RT-PCR

Total RNA was extracted from ∼30 embryos or from P19CL6 cells cultured in 6-well plates to 80-90% confluence at using the RNeasy mini kit. After DNase treatment, 1 µg of total RNA was retro-transcribed using miScript Reverse Transcription kit (for miRNA analysis) and Quantitec Reverse Transcription kit (for gene analysis) following the manufacturer’s instruction. Real-time PCR (qRT-PCR) was carried out using either QuantiFast SYBR Green kit with Rotor gene (Quiagen) or LightCycler 480 SYBR Green I Master with LightCycler 480 (Roche) following the manufacturer’s instructions. Primers used for mRNA analysis were as follows: for mmu-Tbx5 F 5′-CCACTGGATGCGACAACTT-3′, R5′-GCATGGAGTTCAGGATA ATGTG-3′; for mmu-Slit2 F 5′-GCCACCTCAGTGAGAACCTC-3), R 5′-TGTCCCTCAAAGCCCAGA-3′; for mmu-Slit3 F 5′-GCCACCTCAGTGAGAACCTC-3′, R 5′-TGTCCCTCAAAGCCCAGA-3′; for mmu-Cx40 F 5′-GAGCCTGAAGAAGCCAACT-3′, R 5′-AGCTCCAGTCACCCATCTTG-3′; for mmu-αMHC F 5′-CAAGACTGTCCGGAATGACA-3′, R 5′-GGCTTCTTGTTGGACAGGAT-3′; for mmu-GATA4 F 5′-GGAAGACACCCCAATCTCG-3′, R 5′-CATGGCCCCACAATTGAC-3′; for β III-Tubulin F 5′-TTCTGGTGGACTTGGAACCT-3′, R5′-ACTCTTTCCGCACGACATCT-3′; for mmu-Oct4 F 5′-TCAG CTTGGGCTAGAGAAGG-3′, R 5′-GGCAGAGGAAAGGATACAGC-3′and for mmu-PAX6 F 5′-CCT CCTTCACATCAGGTTCC-3′, R 5′-CATAACTCCGCCCATTCACT-3′. For dre-Tbx5a F 5′-AACCATCT GGATCCCTTCG-3′, R5′-TGTTTTCATCCGCCTTGAC-3′; for dre-Slit2 F 5′-TCCTTTCAAGGTCTCCG TTC-3′, R 5′-AGGCCTTTGGGAAGTTCAGT-3′. Transcript values were normalized with those obtained from the amplification of mmu-α-actin with the following primers: F (5′-CGAGCTGTCTTCCCATCCA-3′), R (5′-TCACCAACGTAGCTGTCTTTCTG-3′) for P19CL6 analysis and from the amplification of dre-α-actin or dre-EF1 respectively with the following primers F 5′-CGAGCTGTCTT CCCATCCA-3′, R 5′-TCA CCAACGTAGCTGTCTTTCTG-3′ and F 5′-CTGGAGGCCAGCTCAAACAT-3′, R 5′-ATCAAGAAGAG TAGTACCGCTAGCATTAC-3′ for zebrafish embryos analysis. The primers for mature *miR-218*a, mmu- or dre-U6 (as standard) quantification were respectively: 5′-TTGTGCTTGATCTAACCATGT-3′, 5′-CGCAAG GATGACACGCAAATTC-3′, 5′-ATGACACGCAAATCCGTGAAG-3′. To amplify both pre-miRNAs of 218 in mouse, the following primers were used: for pre-miR-218-1 F 5′-GATAATGGAGCGAGATTTTCT G-3′, R 5′-TAGAAAGCTGCGTGACGTTC-3′ and for pre-miR-218-2 F 5′-AGTTGCCGCGGGGCTTTC-3′, R 5′-AGGAGAGAGCGATGCTTTC-3′.

All reactions were performed in triplicate. Relative quantification of gene expression was calculated as described [52].

### Dre-tbx5a Cloning

Cardiac tissue-enriched total RNA extracted from 5 day old embryos was used to clone zebrafish *tbx5a*. *dre-tbx5a* cDNA was directely amplified by RT-PCR (forward primer 5′-AGATCTAGAC ATCGTACAGGC-3′, reverse primer 5′-CTGCATGTTAGCTGGCTTCGT-3′) The *tbx5a* PCR fragment was cloned into the pGEM-T Easy Vector (Promega) and sequenced by Genechron C.R.ENEA (Roma, Italy). For zebrafish *tbx5a* mRNA production the entire open reading frame was subcloned in pCS2-GFP vector using BamHI and XbaI restriction sites. The construct was linearized with NotI and the capped mRNA was synthetized with mMESSAGE mMACHINE® Kit (Applied Biosystems). As control, capped mRNA of GFP obtained from pCS2-GFP linearized template was used.

### Morpholinos

The morpholino antisense oligonucleotide MO-Tbx5a (5′-GAAAGGTGTCTTCACTGTCCGCCAT-3′) and MO-Tbx5b (5′-GGATTCGCCATATTCCCGTCTGAGT-3′) were designed against the translational start sites of *tbx5a* and *tbx5b* respectively. To test the specificity of the two MOs two plasmids pCS2-Tbx5a-GFP and pCS2-Tbx5b-GFP were generated by fusing a 180-bp fragment of *tbx5a* and a 219-bp fragment of *tbx5b*, both including the morpholino target sides, into GFP producing vector. The primers used for fragment amplifications were: for Tbx5a F 5′-AAGCTTCAACCGCTAGTGCTGGAAG-3′, R 5′-GGATCC TTCGCTGTCACTGGGAGAG-3′ for Tbx5b F 5′-AAGCTTGAGAGCCGGAGAAGACTG-3′, R 5′-GGATCCCATTGGATTCGCCATATTC-3′ where the underlined sequences represent anchored Hind III (F primers) and Bam HI (R primers) restriction sites used for subcloning in pCS2 vector. *tbx5a* and *b* PCR fragments were cloned in pGEM-T Easy Vector, sequenced and subcloned in pCS2-GFP vector.

The morpholino antisense oligonucleotide MO^M^-218 (5′-GCACATGGTTAGATCAAGCACAACA-3′) and MO^D^-218 (5′-TGCATGGTTAGATCAAGCACAAGGG-3′) were designed against the mature form of *miR-218*a and the Drosha cleavage site of pre-*miR-218*a respectively. The sequence of the control MO was 5′-CCTCTTACCTCAGTTACAATTTATA-3′.

### Microinjection

The one- and two-cell stage embryos were injected with a constant injection volume (∼1 nl, confirmed by volume analysis) using a microinjector (Tritech Research, Los Angeles CA USA).

### Optical Microscopy and Confocal Analysis

Optical microscopy was performed with Olympus SZH microscope, images were acquired with Nikon DS-Fi1 camera and NIS-Elements F 3.0 software. Images were processed with Gimp-2.6 software. For confocal analysis embryos were fixed in 4% PFA for 1 h at room temperature under slow agitation and embedded in 2% low-melt agarose. Images were acquired with a Leica DM IRE 2 confocal microscope. Image stacks were processed with FIJI-WIN32 by projection. For migration analysis, embryos were injected with the indicated miRNA mimics or morpholinos and allowed to develop at 26°C until the 14-somite stage. Then they were embedded in 1% low-melting soft agarose and imaged at 26°C with Leica DM IRE 2 confocal microscope to obtain stacks. To calculate cell velocity stacks were processed with MATLAB.

### FACS Analysis

Zebrafish *Tg(tie-2:GFP-cmlc2:eRFP)* embryos were injected and raised in standard conditions until 72 hpf. Next embryos (n = 15−25 embryos for each analysis) were collected and treated with trypsin 0,125 mg/l at 37°C. After a 30 min incubation, trypsin was inactivated, samples were filtered with 50 µm cell-strainer and processed with FACScalibur BD.

### Whole Mount ***in situ*** Hybridization

*slit2* and *slit3* clones for *in situ* hybridization were kindly provided by Dr. Hitoshi Okamoto (Laboratory for Development of Gene Regulation, RIKEN, Brain Science Institute Japan).

Whole mount *in situ* hybridization was performed as previously described [53] with some modification: pre-hybridization temperature was 62°C; hybridization temperature was 62°C for gene probes and 52°C for *miR-218* probe. 500 ng of antisense DIG labelled RNA probe was added to the hybridization mix. The anti-DIG antibody-alkaline phosphatase Fab fragment was diluted 1∶4.000 in MABlock buffer (2% Blocking reagent in 100 mM Maleic acid and 150 mM NaCl) and incubation was performed at +4°C for gene probes and at room temperature for *miR-218* probe. After incubation with the anti-DIG antiserum, embryos were washed in PBT and then incubated in the alkaline Tris buffer solution containing 2 mM Tetramisole (Sigma). The final staining step was performed in BM Purple AP-Substrate, precipitating buffer according to the manufacturer’s recommendations. Labeled embryos older than 24 h were bleached as follows: embryos were washed at room temperature under slow agitation in PBS changed 2x at 5 min. intervals. PBS was removed and embryos were incubated for 10 minutes in pre-bleaching buffer (SSC 0,5X and 6% Formamide). After discarding the pre-bleaching buffer, bleaching buffer (pre-bleaching buffer containing 33% H_2_O_2_) was added to the embryos which were subsequently exposed to a bright source of light for 15 minutes. Afterwards embryos were transferred back to 87% glycerol in PBS incubated at room temperature under slow agitation for 4 h and then stored at –20°C.

### Statistical Analysis

Data were analyzed using GraphPad Prism (GraphPad Software, San Diego, CA USA). Statistical differences were determined by unpaired *t*-test, with values of *P*<0.05 considered statistically significant. Each experimental point in the graph represents the mean ± SE of at least three independent experiments. In the graph of Fig. 6A comparisons between groups were performed by one-way analysis of variance, followed by Bonferroni’s post-hoc for multiple comparisons.

## Supporting Information

Figure S1 **Schematic representation of the bioinformatic tool developed to identify in silico Tbx5-controlled miRNAs.** Ensembl was used to obtain information about chromosome location, position and segment of the selected genes. Ensembl is a joint scientific project between the European Bioinformatics Institute (EBI; http://www.ebi.ac.uk/clustalw/) and the Wellcome Trust Sanger Institute (http://www.sanger.ac.uk/). EBI provides a centralized resource with annotations on genomes of sequenced species and the Ensembl Perl API (Application Programming Interface) models for access to biological objects, such as genes and proteins. Moreover EBI allows the execution of Perl programs for retrieving data from a public database MySQL (http://www.mysql.it/). We generated two local databases, one for genes, and one for microRNAs. By applying the Perl program that uses Ensembl API, we compared the gene databases with the microRNA database using a standard database interface module for Perl.
(TIF)

Figure S2 **tbx5 and miR-218 are co-expressed in mouse tissues.** A-D, relative expression of *tbx5*, *slit2*, *slit3* and *miR-218* as evaluated by q-RT-PCR in different newborn mouse tissues. Results are standardized against GAPDH for genes, and against U6 for miRNAs. Values represent the averages and standard deviations of at least two independent experiments.
(TIF)

Figure S3 **Expression of pre-miR-218-1 parallels that of mature miR-218 during mouse differentiation and tbx5 modulation.** Q-RT-PCR detection of *pre-miR-218-1* and *pre-miR-218-2* relative expression in P19CL6 cells during differentiation (A), or 48 h after plasmids or siRNA transfection (B). In B fold changes of CMV-Tbx5 and siRNA-Tbx5 are relative to CMV-empty and siRNA-Ct values, respectively. Results are standardized against GAPDH. *, P<0.05 (Student’s t-test). C, Q-RT-PCR detection of *slit2* and mature *miR-218* in P19CL6 cells transfected with a mix of two siRNAs against *slit2* or with a siRNA-Ct.
(TIF)

Figure S4 **Rescue of cardiac defects induced by miR-218a over-expression was accomplished by co-injecting MO-218a.** A, qRT-PCR analysis of *miR-218a* relative expression in 24 hpf embryos microinjected with 12 ng of control morpholino (MO-Ct) or MO^D^-218a and with 260 pg of miR-Ct or *miR-218a* mimic. *miR-218a* relative expression was calculated as the ratio between the expression of injected and the expression of non injected embryos. B, representative transgenic *Tg(cmlc2:eGFP)* embryos at 72 hpf showing heart morphological defects induced by the injection of 260 pg of *miR-218a* mimic in the absence (a,b) or in the presence (c,d) of MO^D^-218a (12 ng). Labels: a, atrium, v, ventricle. Black scale bars: 100 µm, red scale bars 25 µm.
(TIF)

Figure S5 **miR-218 dysregulation does not affect vascular integrity.** Confocal images of representative 72 hpf *Tg(flk1:eGFP)* embryos injected with 260 pg of miR-Ct (A), 260 pg of miR-218 mimic (B) or 8 ng MO^D^-218 (C). Black and white scale bars: 100 µm.
(TIF)

Figure S6 **tbx5 and miR-218a misexpression does not alter amhc and vmhc cardiac marker expression in zebrafish embryos.** Ventral views of 48 hpf embryos injected with the indicated miRNA mimics (260 pg) or MOs (12 ng MO-Ct and MO^D^-218a, 3 ng MO-Tbx5a) after mRNA *in situ* hybridization. Scale bar 100 µm.
(TIF)

Figure S7 **miR-218 targets the 3′ UTR of robo1 in zebrafish embryos.** Top: schematic representation of sensors and miRNAs used for *in vivo* sensor assay. Bottom: examples of 24 hpf embryos microinjected with 40 pg of RFP mRNA, 400 pg of 3′UTR *robo 1* sensor and 160 pg of *miR-Ct* (a,c,e) or *miR-218a* (b,d,f). In figures C and D the percentage of the relative phenotypes were indicated. ∼30 embryos for each thesis were injected. Scale bars 50 µm.
(TIF)

Figure S8 **MO-Tbx5a and MO-Tbx5b effectively knockdown the two zebrafish tbx5 isoforms.** A-D, 35 pg of pCS2 plasmid expressing GFP fused with MO-Tbx5a or MO-Tbx5b target sequences were injected in one-cell stage embryos in the absence (A and C) or in the presence (B and D) of 1,5 ng of the relative morpholino. Representative fluorescent images of 24 hpf embryos. ∼20 embryos for each thesis were analysed. E-F, Tbx5 morphants analysis. Phenotypic analysis of Tbx5a (E) and Tbx5b (F) morphants: 2 ng of MO-Tbx5a, or 4 ng of MO-Tbx5b, were injected in *Tg(cmlc2:eGFP)* embryos. Phase-contrast images showing pericardial edema (arrowheads) and fin absence (brackets) or presence (arrows); in the bottom right corner of figures E and F, fluorescent images showing heart morphology. Quantification of Tbx5a (G) and Tbx5b (H) morphant phenotypes. The percentage of embryos with the indicate defects was averaged across multiple independent experiments. ∼100 embryos for each thesis were analysed. Black scale bars: 100 µm, red scale bars 25 µm.
(TIF)

Figure S9 **Injection of miR-218a in Tbx5a morphants increases the severity of heartstring phenotype.** A, phenotypic analysis of Tbx5a morphants co-injected with 1 ng of MO-Tbx5a and either 130 pg of *miR-218a* mimic or 130 pg of miR-Ct. B, representative images of 48 hpf embryos showing the edema expansion caused by the co-injection of *miR-218a* mimic. C, phenotypic analysis of embryos co-injected with sub-phenotypic doses of both MO-Tbx5a(0.5 ng) and *miR-218a* mimic (35 pg). For comparison the same dose of MO-Tbx5a was co-injected with 35 pg of *miR-Ct*. (D-E) Representative confocal images showing heart morphology of transgenic *Tg(cmlc2:eGFP)* embryos injected with a sub-phenotypic dose of MO-Tbx5a and 35 pg of either *miR-Ct* (D) or *miR-218a* mimic (E). Embryo in D has normal looping while co-injected embryos in E show absence of looping, although with different degrees of heart defects. *a*, atrium, *v*, ventricle, *e*, cardiac edema. Black and white scale bars: 100 µm, red scale bars 25 µm.
(TIF)
