# Supplementary figures and images for: MicroRNA 218 Mediates the Effects of Tbx5a Over-Expression on Zebrafish Heart Development
Source: PLoS One. 2012 Nov 30;7(11):e50536. doi: 10.1371/journal.pone.0050536 (PMC3511548; doi:10.1371/journal.pone.0050536)

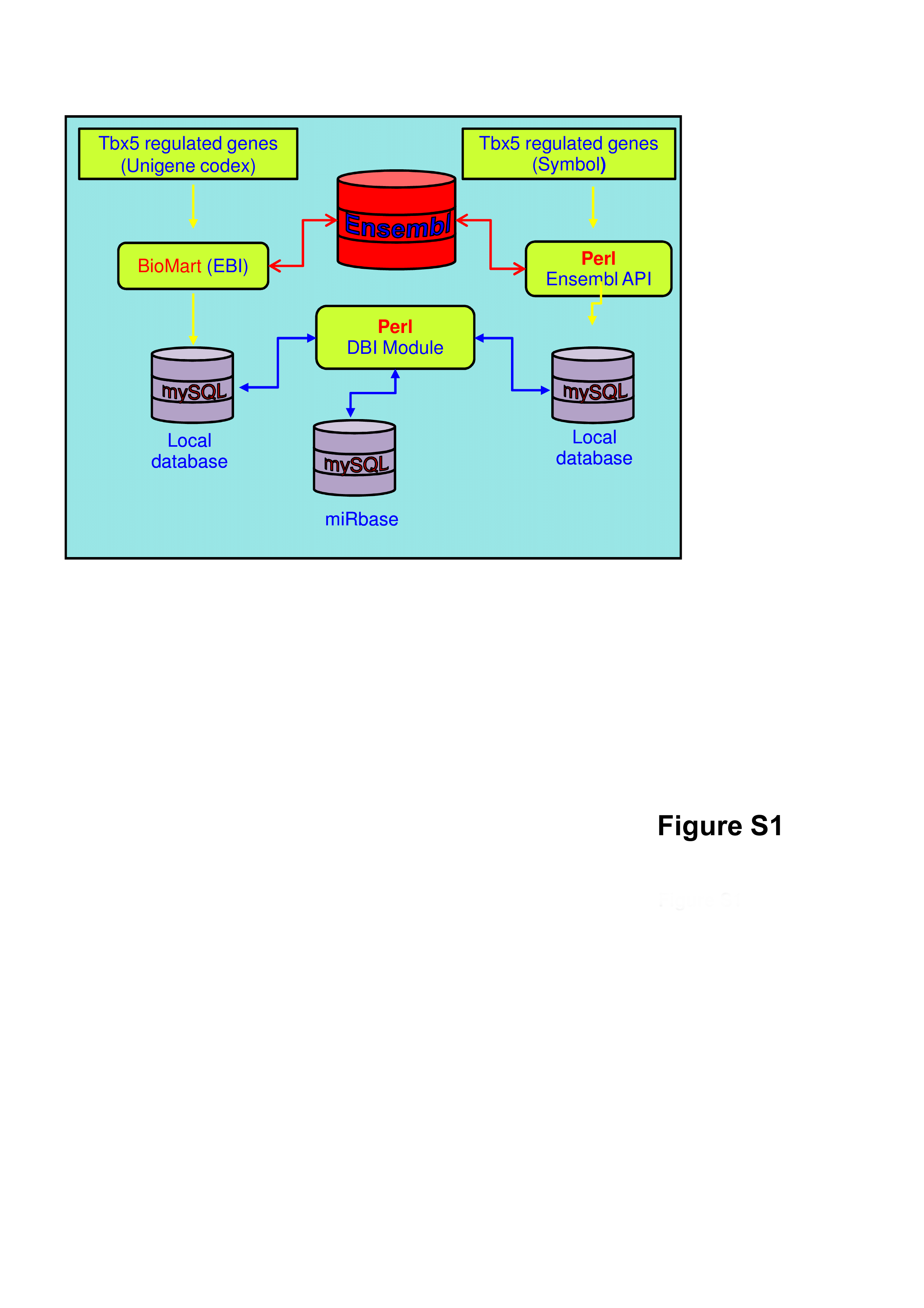

Supplement: Figure S1 — Schematic representation of the bioinformatic tool developed to identify in silico Tbx5-controlled miRNAs. Ensembl was used to obtain information about chromosome location, position and segment of the selected genes. Ensembl is a joint scientific project between the European Bioinformatics Institute (EBI; http://www.ebi.ac.uk/clustalw/) and the Wellcome Trust Sanger Institute (http://www.sanger.ac.uk/). EBI provides a centralized resource with annotations on genomes of sequenced species and the Ensembl Perl API (Application Programming Interface) models for access to biological objects, such as genes and proteins. Moreover EBI allows the execution of Perl programs for retrieving data from a public database MySQL (http://www.mysql.it/). We generated two local databases, one for genes, and one for microRNAs. By applying the Perl program that uses Ensembl API, we compared the gene databases with the microRNA database using a standard database interface module for Perl. (TIF) [file pone.0050536.s001.tif]

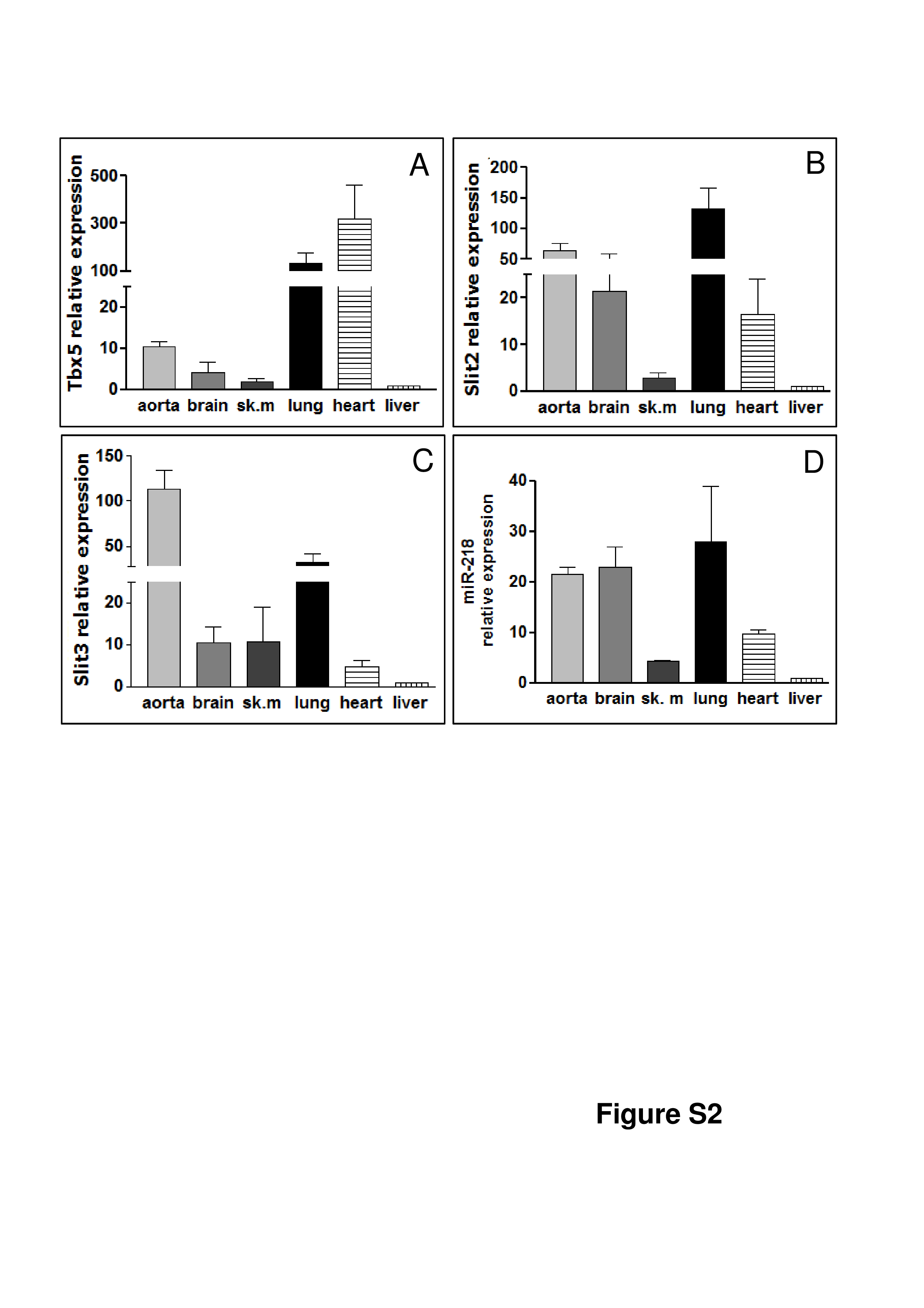

Supplement: Figure S2 — tbx5 and miR-218 are co-expressed in mouse tissues. A-D, relative expression of tbx5, slit2, slit3 and miR-218 as evaluated by q-RT-PCR in different newborn mouse tissues. Results are standardized against GAPDH for genes, and against U6 for miRNAs. Values represent the averages and standard deviations of at least two independent experiments. (TIF) [file pone.0050536.s002.tif]

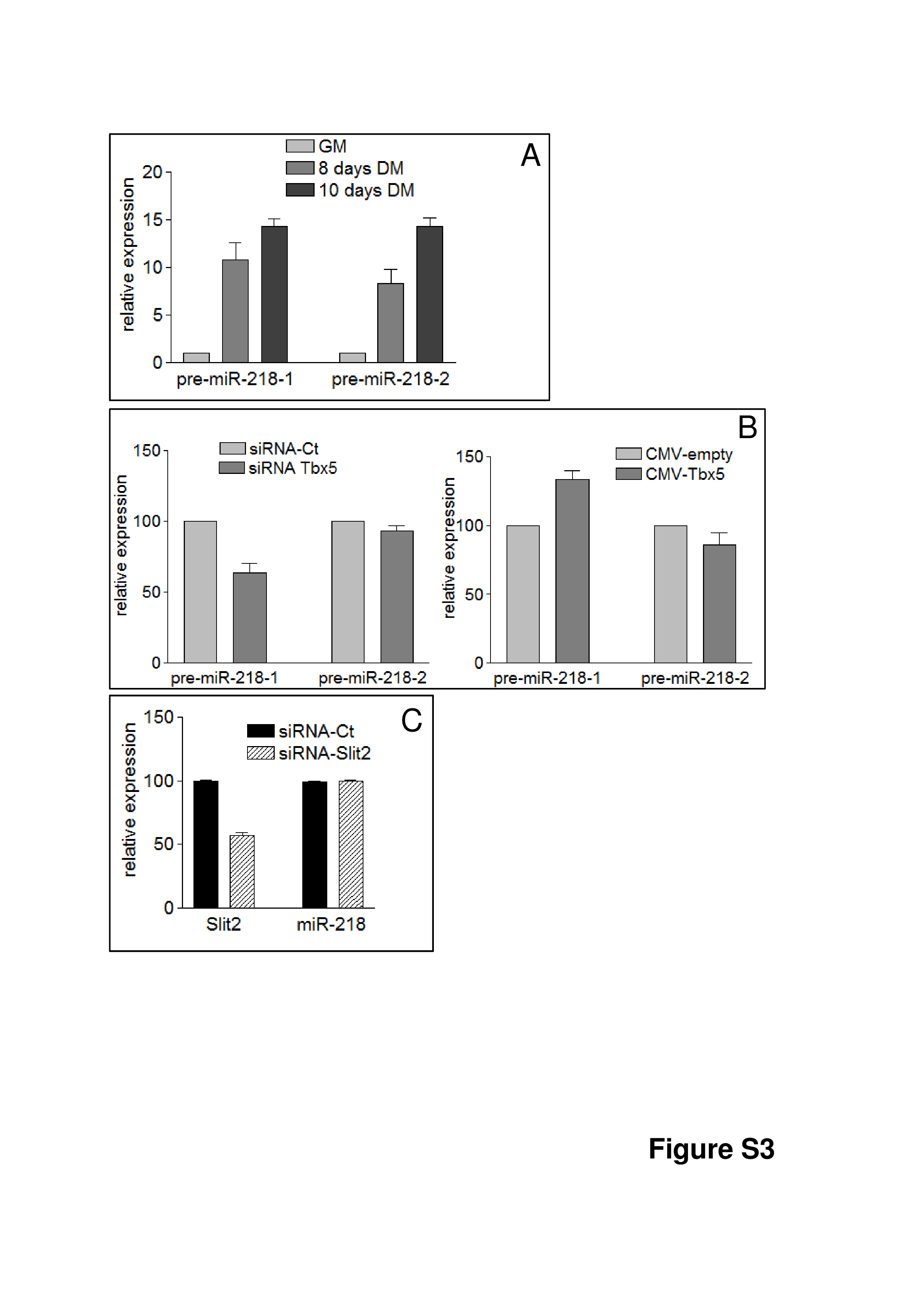

Supplement: Figure S3 — Expression of pre-miR-218-1 parallels that of mature miR-218 during mouse differentiation and tbx5 modulation. Q-RT-PCR detection of pre-miR-218-1 and pre-miR-218-2 relative expression in P19CL6 cells during differentiation (A), or 48 h after plasmids or siRNA transfection (B). In B fold changes of CMV-Tbx5 and siRNA-Tbx5 are relative to CMV-empty and siRNA-Ct values, respectively. Results are standardized against GAPDH. *, P<0.05 (Student’s t-test). C, Q-RT-PCR detection of slit2 and mature miR-218 in P19CL6 cells transfected with a mix of two siRNAs against slit2 or with a siRNA-Ct. (TIF) [file pone.0050536.s003.tif]

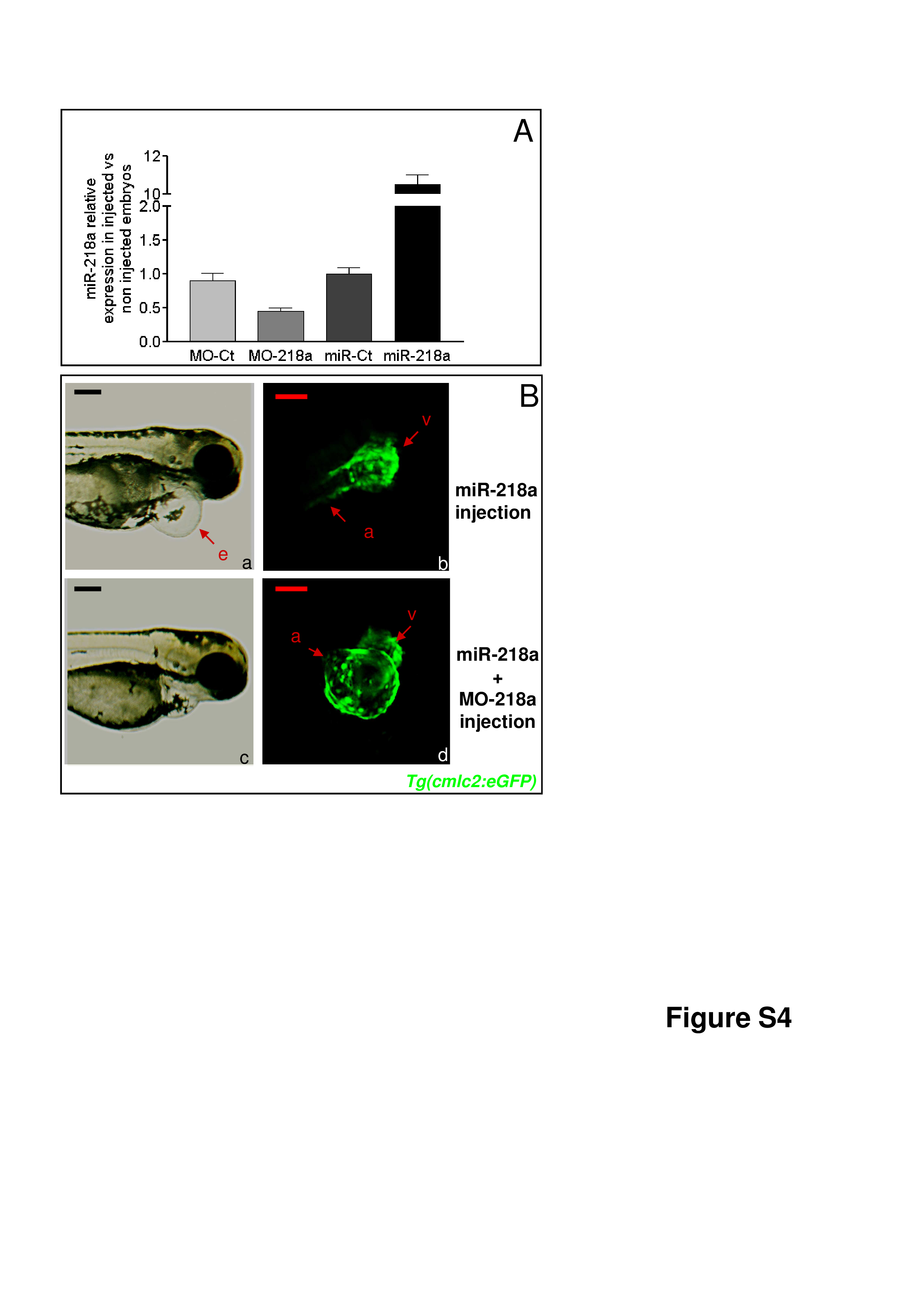

Supplement: Figure S4 — Rescue of cardiac defects induced by miR-218a over-expression was accomplished by co-injecting MO-218a. A, qRT-PCR analysis of miR-218a relative expression in 24 hpf embryos microinjected with 12 ng of control morpholino (MO-Ct) or MOD-218a and with 260 pg of miR-Ct or miR-218a mimic. miR-218a relative expression was calculated as the ratio between the expression of injected and the expression of non injected embryos. B, representative transgenic Tg(cmlc2:eGFP) embryos at 72 hpf showing heart morphological defects induced by the injection of 260 pg of miR-218a mimic in the absence (a,b) or in the presence (c,d) of MOD-218a (12 ng). Labels: a, atrium, v, ventricle. Black scale bars: 100 µm, red scale bars 25 µm. (TIF) [file pone.0050536.s004.tif]

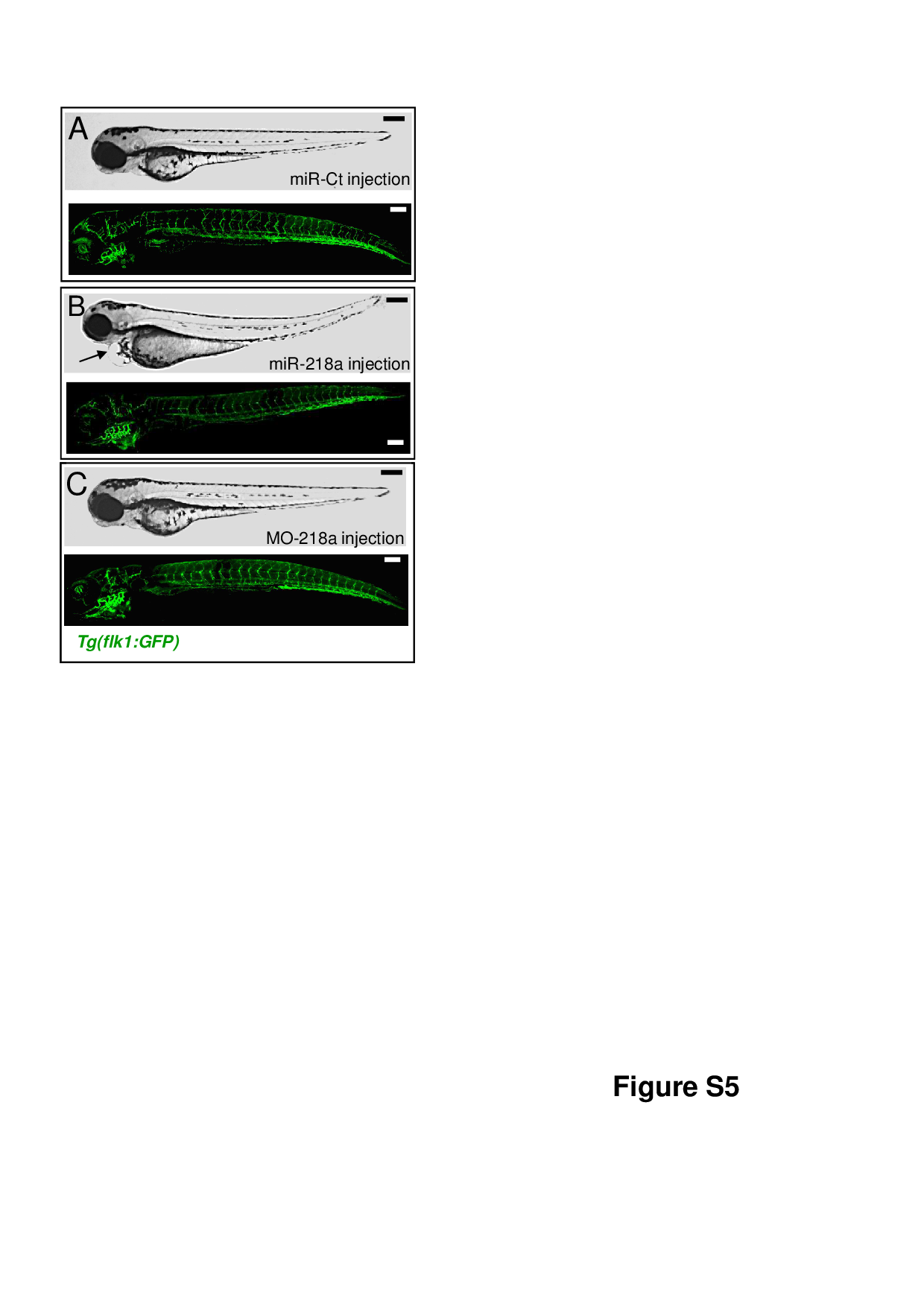

Supplement: Figure S5 — miR-218 dysregulation does not affect vascular integrity. Confocal images of representative 72 hpf Tg(flk1:eGFP) embryos injected with 260 pg of miR-Ct (A), 260 pg of miR-218 mimic (B) or 8 ng MOD-218 (C). Black and white scale bars: 100 µm. (TIF) [file pone.0050536.s005.tif]

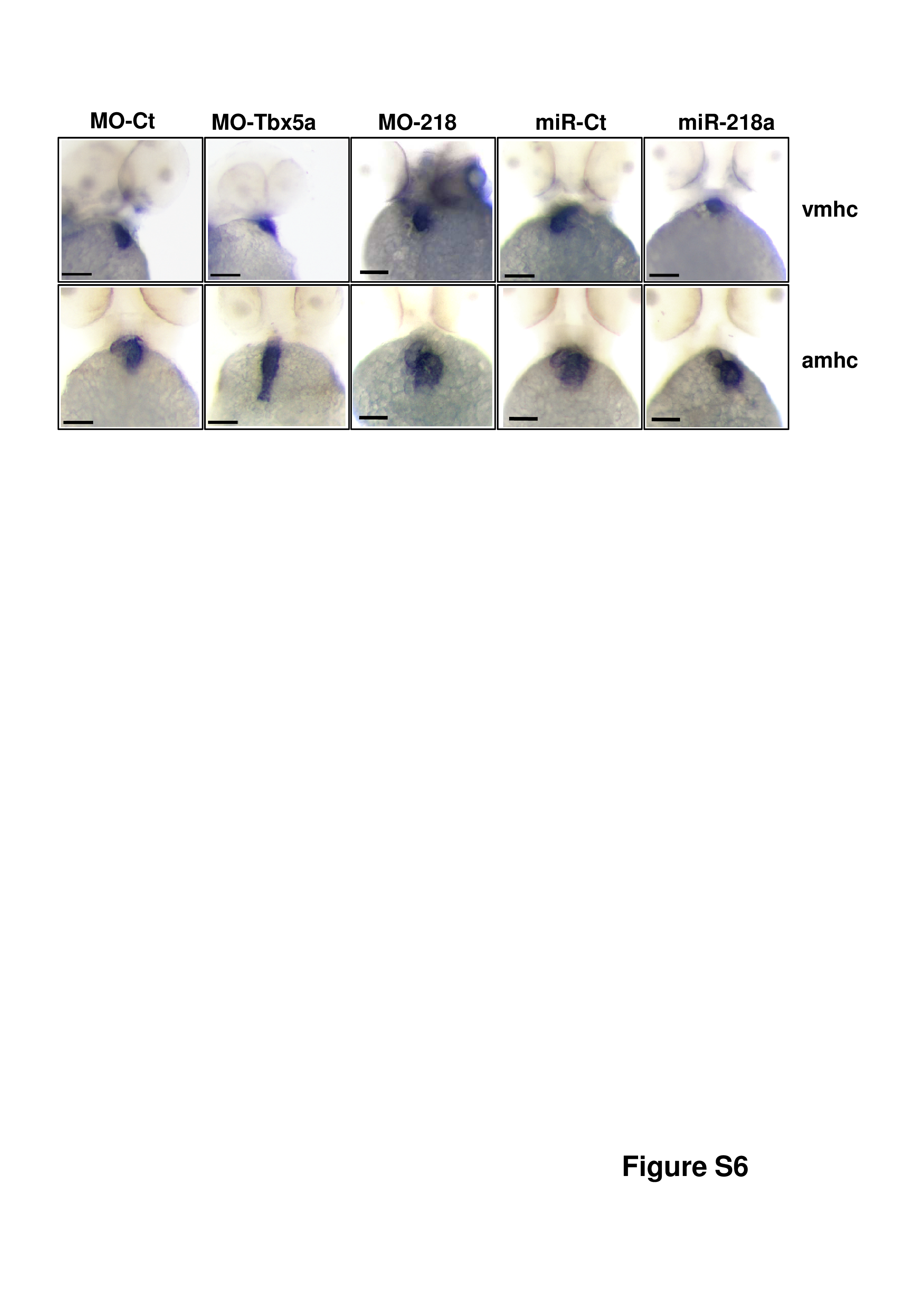

Supplement: Figure S6 — tbx5 and miR-218a misexpression does not alter amhc and vmhc cardiac marker expression in zebrafish embryos. Ventral views of 48 hpf embryos injected with the indicated miRNA mimics (260 pg) or MOs (12 ng MO-Ct and MOD-218a, 3 ng MO-Tbx5a) after mRNA in situ hybridization. Scale bar 100 µm. (TIF) [file pone.0050536.s006.tif]

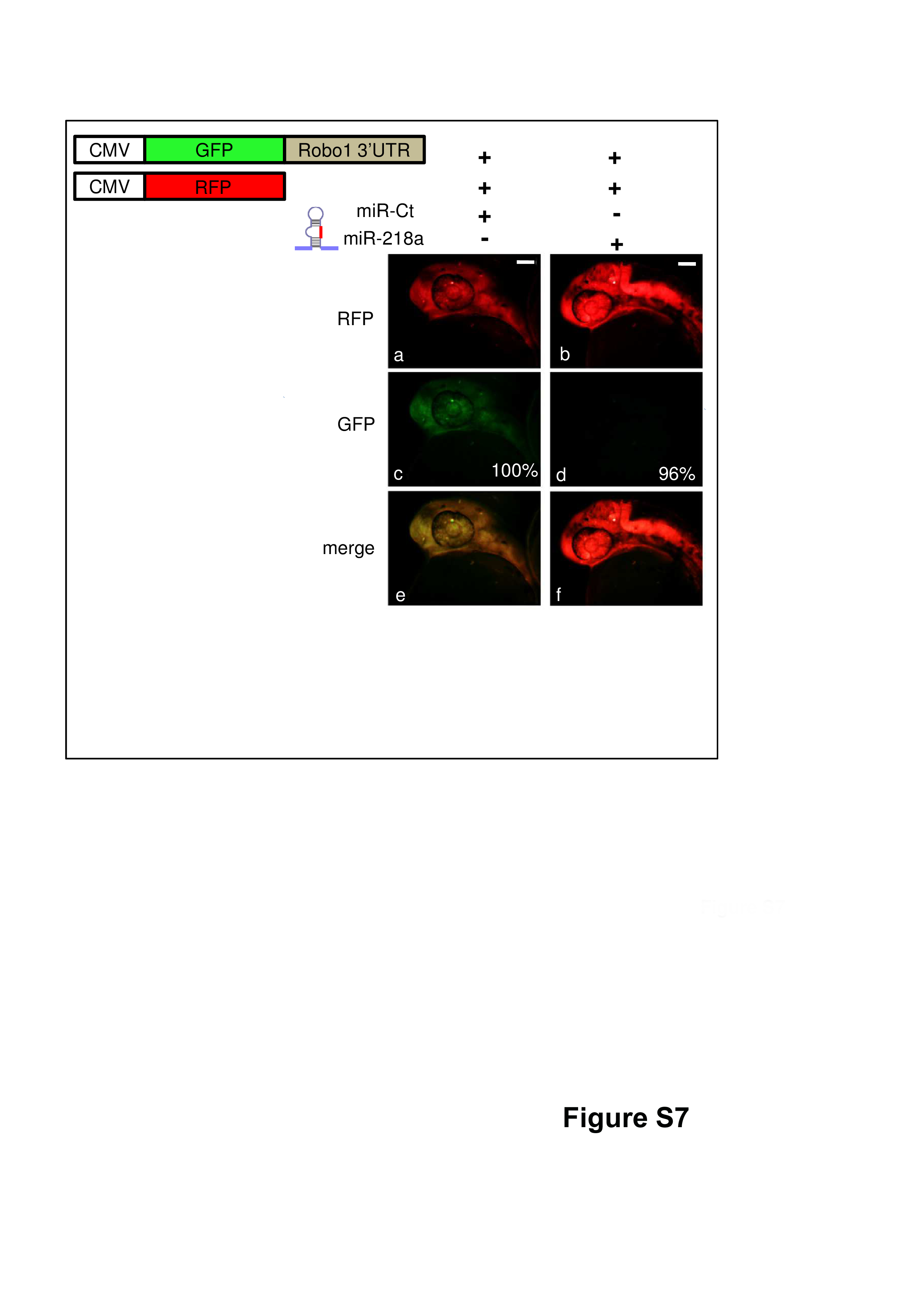

Supplement: Figure S7 — miR-218 targets the 3′ UTR of robo1 in zebrafish embryos. Top: schematic representation of sensors and miRNAs used for in vivo sensor assay. Bottom: examples of 24 hpf embryos microinjected with 40 pg of RFP mRNA, 400 pg of 3′UTR robo 1 sensor and 160 pg of miR-Ct (a,c,e) or miR-218a (b,d,f). In figures C and D the percentage of the relative phenotypes were indicated. ∼30 embryos for each thesis were injected. Scale bars 50 µm. (TIF) [file pone.0050536.s007.tif]

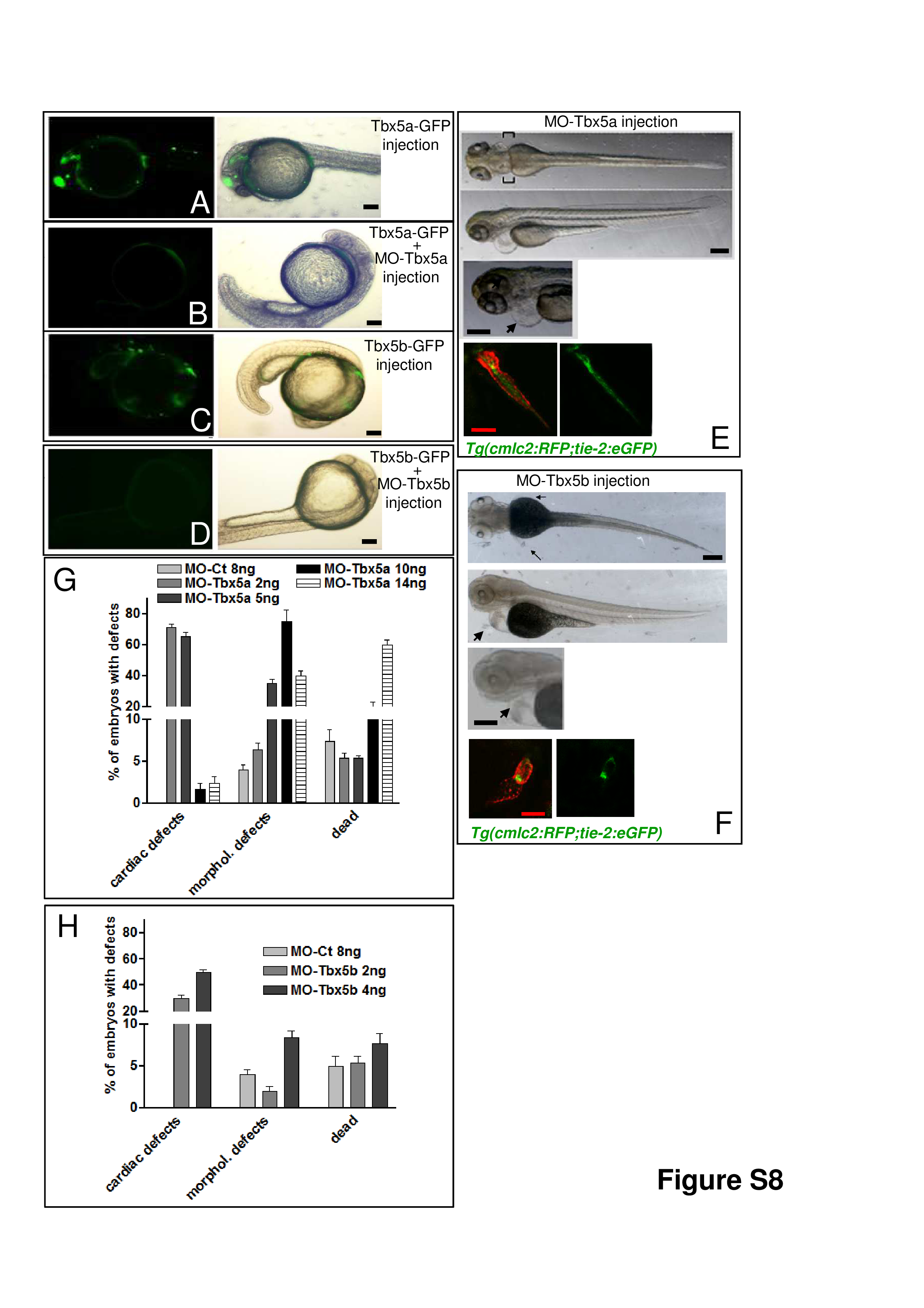

Supplement: Figure S8 — MO-Tbx5a and MO-Tbx5b effectively knockdown the two zebrafish tbx5 isoforms. A-D, 35 pg of pCS2 plasmid expressing GFP fused with MO-Tbx5a or MO-Tbx5b target sequences were injected in one-cell stage embryos in the absence (A and C) or in the presence (B and D) of 1,5 ng of the relative morpholino. Representative fluorescent images of 24 hpf embryos. ∼20 embryos for each thesis were analysed. E-F, Tbx5 morphants analysis. Phenotypic analysis of Tbx5a (E) and Tbx5b (F) morphants: 2 ng of MO-Tbx5a, or 4 ng of MO-Tbx5b, were injected in Tg(cmlc2:eGFP) embryos. Phase-contrast images showing pericardial edema (arrowheads) and fin absence (brackets) or presence (arrows); in the bottom right corner of figures E and F, fluorescent images showing heart morphology. Quantification of Tbx5a (G) and Tbx5b (H) morphant phenotypes. The percentage of embryos with the indicate defects was averaged across multiple independent experiments. ∼100 embryos for each thesis were analysed. Black scale bars: 100 µm, red scale bars 25 µm. (TIF) [file pone.0050536.s008.tif]

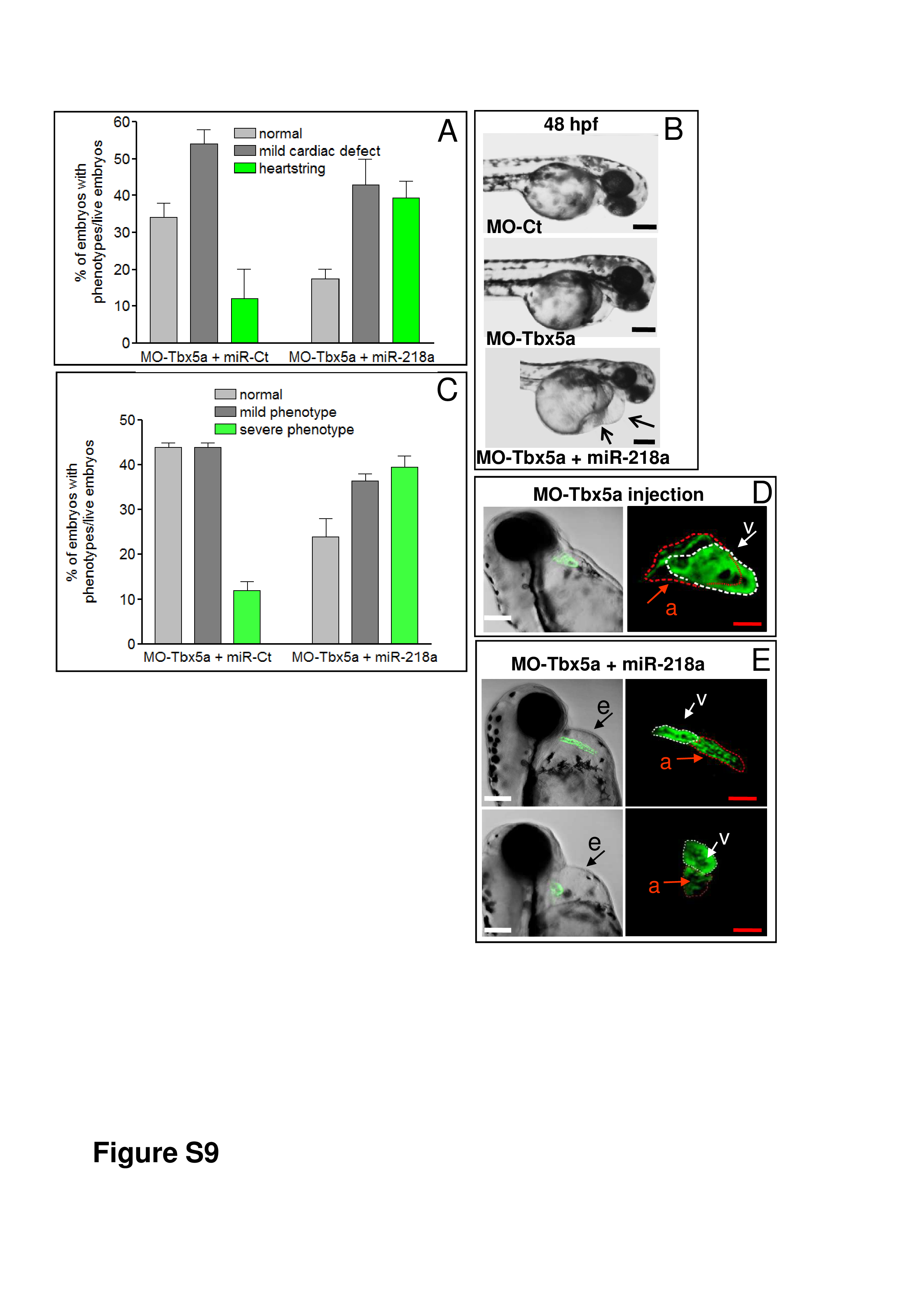

Supplement: Figure S9 — Injection of miR-218a in Tbx5a morphants increases the severity of heartstring phenotype. A, phenotypic analysis of Tbx5a morphants co-injected with 1 ng of MO-Tbx5a and either 130 pg of miR-218a mimic or 130 pg of miR-Ct. B, representative images of 48 hpf embryos showing the edema expansion caused by the co-injection of miR-218a mimic. C, phenotypic analysis of embryos co-injected with sub-phenotypic doses of both MO-Tbx5a(0.5 ng) and miR-218a mimic (35 pg). For comparison the same dose of MO-Tbx5a was co-injected with 35 pg of miR-Ct. (D-E) Representative confocal images showing heart morphology of transgenic Tg(cmlc2:eGFP) embryos injected with a sub-phenotypic dose of MO-Tbx5a and 35 pg of either miR-Ct (D) or miR-218a mimic (E). Embryo in D has normal looping while co-injected embryos in E show absence of looping, although with different degrees of heart defects. a, atrium, v, ventricle, e, cardiac edema. Black and white scale bars: 100 µm, red scale bars 25 µm. (TIF) [file pone.0050536.s009.tif]
